# *Cordyceps* spp.: A Review on Its Immune-Stimulatory and Other Biological Potentials

**DOI:** 10.3389/fphar.2020.602364

**Published:** 2021-02-08

**Authors:** Gitishree Das, Han-Seung Shin, Gerardo Leyva-Gómez, María L. Del Prado-Audelo, Hernán Cortes, Yengkhom Disco Singh, Manasa Kumar Panda, Abhay Prakash Mishra, Manisha Nigam, Sarla Saklani, Praveen Kumar Chaturi, Miquel Martorell, Natália Cruz-Martins, Vineet Sharma, Neha Garg, Rohit Sharma, Jayanta Kumar Patra

**Affiliations:** ^1^ Research Institute of Biotechnology and Medical Converged Science, Dongguk University-Seoul, Goyangsi, South Korea; ^2^ Department of Food Science and Biotechnology, Dongguk University-Seoul, Goyangsi, South Korea; ^3^ Departamento de Farmacia, Facultad de Química, Universidad Nacional Autónoma de México, Ciudad de México, Mexico; ^4^ Laboratorio de Medicina Genómica, Departamento de Genética, Instituto Nacional de Rehabilitación Luis Guillermo Ibarra Ibarra, Ciudad de México, Mexico; ^5^ Department of Post-Harvest Technology, College of Horticulture and Forestry, Central Agricultural University, Pasighat, India; ^6^ Environment and Sustainability Department, CSIR-Institute of Minerals and Materials Technology, Bhubaneswar, India; ^7^ Adarsh Vijendra Institute of Pharmaceutical Sciences, Shobhit University, Saharanpur, India; ^8^ Department of Biochemistry, H. N. B. Garhwal University, Srinagar Garhwal, India; ^9^ Department of Pharmaceutical Chemistry, H. N. B. Garhwal University, Srinagar Garhwal, India; ^10^ Department of Nutrition and Dietetics, Faculty of Pharmacy, and Centre for Healthy Living, University of Concepción, Concepción, Chile; ^11^ Faculty of Medicine, Alameda Prof. Hernani Monteiro, University of Porto, Porto, Portugal; ^12^ Institute for Research and Innovation in Health, University of Porto, Porto, Portugal; ^13^ Laboratory of Neuropsychophysiology, Faculty of Psychology and Education Sciences, University of Porto, Porto, Portugal; ^14^ Department of Rasa Shastra and Bhaishajya Kalpana, Faculty of Ayurveda, Institute of Medical Sciences, Banaras Hindu University, Varanasi, India; ^15^ Department of Medicinal Chemistry, Faculty of Ayurveda, Institute of Medical Sciences, Banaras Hindu University, Varanasi, India

**Keywords:** ethnopharmacology, cordyceps, cordycepin, natural medicine, immune system, immunostimulation, immunomodulatory, clinical studies

## Abstract

In recent decades, interest in the *Cordyceps* genus has amplified due to its immunostimulatory potential. *Cordyceps* species, its extracts, and bioactive constituents have been related with cytokine production such as interleukin (IL)-1β, IL-2, IL-6, IL-8, IL-10, IL-12, and tumor necrosis factor (TNF)-α, phagocytosis stimulation of immune cells, nitric oxide production by increasing inducible nitric oxide synthase activity, and stimulation of inflammatory response via mitogen-activated protein kinase pathway. Other pharmacological activities like antioxidant, anti-cancer, antihyperlipidemic, anti-diabetic, anti-fatigue, anti-aging, hypocholesterolemic, hypotensive, vasorelaxation, anti-depressant, aphrodisiac, and kidney protection, has been reported in pre-clinical studies. These biological activities are correlated with the bioactive compounds present in *Cordyceps* including nucleosides, sterols, flavonoids, cyclic peptides, phenolic, bioxanthracenes, polyketides, and alkaloids, being the cyclic peptides compounds the most studied. An organized review of the existing literature was executed by surveying several databanks like PubMed, Scopus, etc. using keywords like *Cordyceps*, cordycepin, immune system, immunostimulation, immunomodulatory, pharmacology, anti-cancer, anti-viral, clinical trials, ethnomedicine, pharmacology, phytochemical analysis, and different species names. This review collects and analyzes state-of-the-art about the properties of *Cordyceps* species along with ethnopharmacological properties, application in food, chemical compounds, extraction of bioactive compounds, and various pharmacological properties with a special focus on the stimulatory properties of immunity.

## Introduction

The fungus *Cordyceps* spp. belongs to Tibetan medicine and consumers describe it as an important source of energy. *Cordyceps* spp. belongs to Ascomycota, Pyrenomycetes, Hypocreales, and Clavicepitaceae, and at least 700 species are known. The word *Cordyceps* originates from the Greek term “kordyle”, which means “club”, and the Latin etymon “ceps”, which means “head” (Olatunji et al., 2018). *Cordyceps* species invade insects, arthropods, other fungi, and evades the host immune system by harmonizing the life cycle of its host with the intention of survival and multiplication. Their interaction with the host will produce different secondary metabolites (Olatunji et al., 2018), such as cordycepin, adenosine, guanosine, cordymin, γ-aminobutyric acid (GABA), exopolysaccharides, cordysinin A-E, among others (Liu Y et al., 2015).

The different species of *Cordyceps* have beneficial properties such as anti-cancer, anti-proliferative, anti-angiogenic, anti-metastasis, apoptosis induction, anti-inflammatory, antioxidant, anti-fibrotic, anti-arteriosclerosis, anti-hypertensive, anti-thrombotic, antimalarial, antifungal, hypolipidemic, antidiabetic, hypoglycemic, anti-asthmatic, steroidogenesis, spermatogenic, anti-aging, and immunomodulatory effects (Liu Y et al., 2015). These properties are concentration-dependent, and in most cases, no adverse effects were reported, although the evaluation of isolated compounds such as cordycepin is preferred.

Interestingly, *Cordyceps* spp. contains different compounds with the ability to strengthen the response of the immune system and also to control its exacerbated response. Most of the information on the effect of Cordyceps on the immune system derives from studies in cancer. In particular, *Cordyceps* spp. increases the production of interleukin (IL)-1β, IL-2, IL-6, IL-8, IL-10, IL-12, and tumor necrosis factor (TNF)-α, induces phagocytosis of macrophages, mononuclear cells, nitric oxide (NO) release, and stimulates the inflammatory response via the mitogen-activated protein kinase (MAPK) route (Lee et al., 2006; Wang M et al., 2012). Furthermore, it presents synergism with interferon (INF)-γ in the production of cytokines. These properties are attractive in the search for new applications where the stimulation in the immune system response is wanted. Therefore, this review collects and analyzes the state-of-the-art about properties of *Cordyceps* spp. focused on the stimulatory properties of immunity.

## Methodology

An organized review of the existing literature was executed by surveying pertinent peer-reviewed research articles, review articles, etc. from several available bibliographic databanks such as PubMed, SpringerLink, Elsevier journal, Science Direct, Scopus databases, Google search, etc., using keywords and its combination like *Cordyceps*, cordycepin, natural medicine, immune system, immunostimulation, immunomodulatory, pharmacology, anti-cancer, anti-viral, clinical trials, ethnomedicine, pharmacology, phytochemical analysis, and different species names. Usually, the search was carried out in “title, abstract, and keyword” fields. In each search, normally the review articles were omitted, however, in some instances, some important review articles were also considered. Further only articles published in the English language were considered. Articles that were published with only basic ethnobotanical assessment reports which lack substantial proof of the claim were not included in the study.

## Habitat, Distribution, and Characteristics of *Cordyceps* spp.

From the more than 700 species of mushrooms recognized on the genus Cordyceps, around 20 species parasitize on the genus *Elaphomyces*, meanwhile the remaining species do on insects and arthropods belonging to *Arachnida, Hymenoptera, Isoptera, Coleoptera, Hemiptera,* and *Lepidoptera* classes. This diversity of species includes the *C. sinensis* (*Ophiocordyceps sinensis* (Berk.) G.H.Sung, J.M.Sung, Hywel-Jones and Spatafora)*, C. ophioglossoides* (*Tolypocladium ophioglossoides* (Ehrh.) Quandt, Kepler & Spatafora)*, C. militaris* (L.) Fr.*, C. gracilis* (Grev.) Durieu & Mont.*, C. sobolifera* (Hill ex Watson)*, C. subsessilis* Petch*, C. gunnii* (Berk.) Berk.*, C. cicadae* S.Z. Shing*, C. tuberculate* (Lebert) Maire*, C. scarabaeicola* Kobayasi*, C. minuta* Kobayasi*, C. myrmecophila* Ces.*, C. canadensis* Ellis & Everh*., C. nutans* Pat.*, C. agriota* A. Kawam.*, C. ishikariensis* M. Zang, D. Liu and R. Hu*, C. sphecocephala* (Berk.) Sacc*, C. konnoana* Kobayasi & Shimizu*, C. nigrella* Kobayasi & Shimizu*, C. pruinosa* Petch*, C. tricentri* Yasuda, among others (Tuli et al., 2013a; Lo et al., 2013; Baral et al., 2015; Pal and Misra, 2018).

These species exhibit different characteristics such as pharmaceutical properties, making them attractive to traditional Chinese medicine (TMC) since the nineties, and being *C. sinesis* the most studied and applied. Their geographic distribution is mainly based on the host distribution; however, they can grow in high mountains at an altitude of 3,600–4,000 m above the sea level. Thus, *Cordyceps* spp. has been found in North America, Europe, and Asia, mostly in countries such as China, Japan, Nepal, Bhutan, Vietnam, Korea, and Thailand. In India, it is principally present in subalpine regions such as Kumaun Himalaya and Garhwal Himalaya (at higher altitudes) (Maity, 2013; Chakraborty et al., 2014). Furthermore, it has been reported that species such as *C. gunnii* (Berk.) Berk*.* was found in Australia (Olatunji et al., 2018). The composition of their metabolite makes them able to tolerate characteristic severe conditions at high altitudes (low temperature, lack of oxygen, and exposure to UV radiation).

On the other hand, the dispersion of this rare and interesting medicinal mushroom is carried out through air, rain, and insects; in its whole life cycle in three phases namely infection, parasitism, and saprophytism (Pal and Misra, 2018). In the first phase, *Cordyceps* spp. infects the host in the larval stage through ascospores, (released in the air from mature fruiting bodies during summer and early autumn), and germinate. In some cases, the infection is produced by the ingestion of food contaminated by *Cordyceps* spp. mycelia. The parasitic stage occurs after the infection, and during this phase, the *Cordyceps* spp. nurtures from the bowel of the host. The fungal cells spread throughout the body and proliferate rapidly during the winter, consuming all internal organs of the larva, leaving intact the exoskeleton. After that, the fungal cell transform into a white mass inside the larva's body (endosclerotium) (Tuli et al., 2013a; Baral et al., 2015). During this process, the environmental conditions are inclement, and the mushroom has to resist the snow and cold conditions. When the spring begins, and the outside temperature increases, the endosclerotium germinates and extrudes through the oral cavity of the host, maturating in summer, forming the fruiting body, and beginning to release ascospores (saprophytic stage). In this season, the fungus collection is carried out.

Traditionally, the primary collectors of these plants are the villagers, who collected it during the time of grazing practice (Baral et al., 2015). During months, the primary gatherers stay in the alpine regions to care for their pet animals (Yak) and collect the fungus and other medicinal plants (Panda, 2010). Local medicine men, who also visit the areas to collect the mushrooms, store the dried material to use it in the future. Due to the medicinal importance of *Cordyceps* spp., its popularity has increased besides the over-harvesting, triggering the scarcity of wild species. For this reason, since the 70s, many scientists have been searching for options to achieve the fermentation and cultivation of fungi isolated. *Cordyceps* spp. have been related to therapeutic properties and healing activities for several years; thus, they have been employed as treatment of different diseases in folk medicine.

## Ethnopharmacy and Traditional Uses of *Cordyceps* spp.

For hundreds of years*, Cordyceps* has been utilized in traditional Chinese medicine (TCM) as a tonic to treat several conditions such as respiratory diseases, liver or renal problems, hyperglycemia, and cancer or tumor disorders. Similarly, *Cordyceps* spp. has been applied as an energy level and endurance enhancer, to improve erobic capacity, and to boost cellular immunity. It was officially classified as a drug in 1964 in Chinese Pharmacopoeia (Shashidhar et al., 2013), being *C. sinensis* and *C. militaris* (L.) Fr. the most frequent species employed.

In some regions such as China, Tibetan plateau, Bhutan, Nepal, and India, the dosage and the administration of *C. sinensis* are dependent on the knowledge and skills of local folk practitioners based on the use of a trial-and-error method (Maity, 2013). For example, some community dissolves the fungus in milk, and alcohol or hot water, to drink it as an enhancer of the desire and sexual potency and as a tonic for the mornings, respectively (Panda and Swain, 2011). The action of the mushroom merged with other bioactive molecules has been reported too. For instance, some folk healers prescribe the use of the *Cordyceps* spp. mixed with taxus leaf and root of ginseng as a cancer treatment.

Furthermore, *C. sinensis* has been described as nutritious food by the Chinese population, probably due to their composition presents nutritional components such as essential amino acids, vitamins (B1, B2, B12, and K), and carbohydrates, among others. Remarkably, this fungus species is a dietary complement that complies with the U.S. Food and Drug Administration (FDA) considerations, which render the cordyceps a demand product in many countries (Wu et al., 2015).

On the other hand, *Cordyceps* spp. has been applied as a remedy for fatigue and weakness, slowing down the symptoms of altitude sickness and giving the patient a boost of energy. At advanced ages, people decrease aches and pains. Similarly, TCM specialists recommend the regular intake of *C. sinensis* to avoid infections, colds, and flues, due to its ability to decrease cough and phlegm, asthma as well as bronchial diseases (Lo et al., 2013). For these reasons, the *Cordyceps* spp. have been applied as a treatment for lung fibrosis, particularly in patients suffering from severe acute respiratory syndrome (SARS). Following the TCM beliefs, all these properties are related to the *C. sinensis* ability to enrich the lung yin and yang (Chiu et al., 2016a). The benefits of *Cordyceps* spp. have also been observed in athletes due to energy improvement derived from the increment of the cellular ATP level, which releases energy in muscle cells.

Similar to *C. sinensis*, the applications of *C. militaris* (L.) Fr. (found in China, Japan, Korea, and East Asia), are related to its properties as an energy enhancer, aphrodisiac source, and respiratory conditions treatment. Besides, hypoglycemic, anti-inflammatory, antitumor, antibacterial, antifungal, antioxidant, and immuno-protective properties have been attributed to this species. Thus, it ranks second in the most commercialized species in China, Japan, and Korea, being considered a suitable cheaper substitute for *C. sinensis* (Chou et al., 2014).

Other species that have been utilized by the folk healers are *C. pruinosa* Petch*, C. bassiana, C. cicadae* S.Z. Shing, C. gunnii (Berk.) Berk.*, C. guangdongensis* T.H. Li, Q.Y. Lin and B. Song*,* and *C. ophioglossoides (T. ophioglossoides)*. The main applications of *C. pruinosa Petch* are in stomach diseases are inflammatory disorders. *C. bassiana* Z.Z. Li, C.R. Li, B. Huang and M.Z. Fan, has been used for skin conditions such as dermatitis and eczema. It also is applied as a biological insecticide for pest control (Wu et al., 2015; Olatunji et al., 2018). In TCM, *C. cicadae* S.Z. Shing has been used to treat infantile convulsions, elevation of temperature, and tremors. Moreover, therapeutic activities such as antitumor, immunoregulatory, and reno-protective have been attributed to this species (Olatunji et al., 2018). Similarly, *C. gunnii* (Berk.) Berk. exhibits immunomodulatory activity, an enhancer effect on the memory, and delay of senescence (Zhu et al., 2012b; Zhu Z-Y et al., 2014). *C. guangdongensis* is employed against fatigue, avian influenza, inflammation, renal failure, and oxidation (Yan et al., 2013). On the other hand, *C. ophioglossoides (T. ophioglossoides)* has been used as food, presenting antitumor, estrogenic, and anti-aging products, besides its application in births to avoid excessive bleeding in women (Kawagishi et al., 2004; Olatunji et al., 2018).

The traditional consumption of *Cordyceps* spp. has been through an herbal product, and its massive marketing dates back to the beginning of the year 2000. In several countries, it is consumed as a food supplement due to its different health attributes. To date, it is a highly sought-after product since its fame increased accompanied by scientific evidence. Prices range up to $20,000 per kilogram for wild *C. sinensis*, making it the most expensive mushroom in the world.

## Chemical Compounds of *Cordyceps* spp.

**FIGURE 1:**
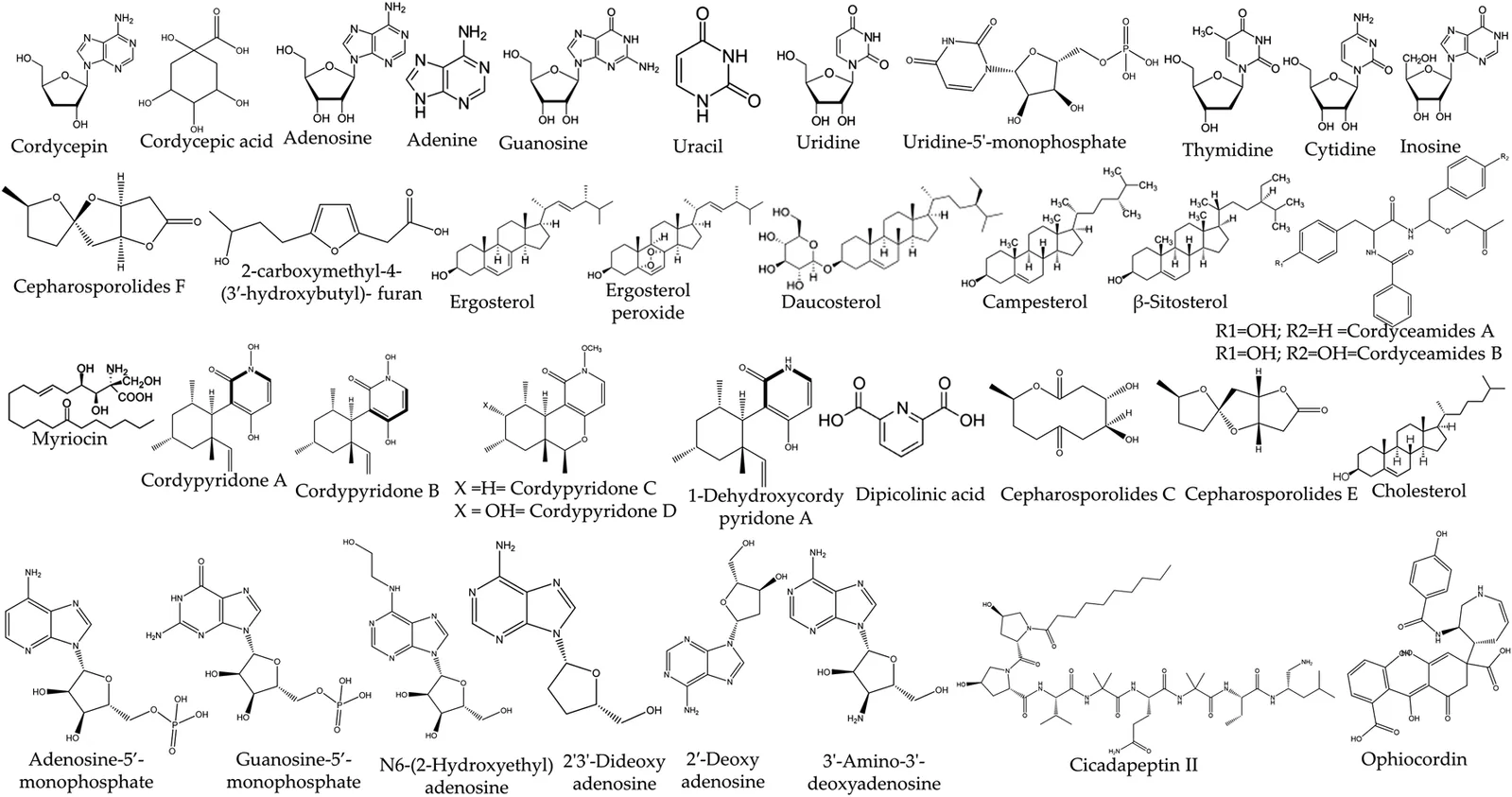
Chemical structure of the bioactive compounds isolated from *Cordyceps* spp.

The genus *Cordyceps* spp. contains a large number of chemical compounds and their derivatives in the form of secondary metabolites. The presence of such diverse chemical compounds makes them quite intriguing in analyzing therapeutic effects and pharmacological studies. Major chemical compounds such as nucleosides, sterols flavonoids, cyclic peptides, phenolic, bioxanthracenes, polyketides, and alkaloids are found in *Cordyceps* species (Table 1, Figure 1). While in most of the *Cordyceps* species, cyclic peptides are present in large quantity as compared to other molecules. Besides that, cordycepin and cordycepic acid (CA) are also prominently present in some species of *Cordyceps spp.* such as *C. militaris* (L.) Fr.*.* The presence of cordycepin (3′-deoxyadenosine) and 2′-deoxyadenosine in *C. sinensis* was characterized by using nuclear magnetic resonance (NMR) and infrared spectroscopy (IR) (Shunzhi and Jingzhi, 1996). In addition to this, a class of saccharides and polysaccharides such as cyclofurans, a cyclic ring of five-carbon sugars, heteropolysaccharides beta-glucans, beta-mannans cross-connected beta-mannan polymers, and complex polysaccharides comprising of both five and six carbon sugars were also discovered from *Cordyceps* spp. Even though, *Cordyceps* spp. contains a lot of bioactive molecules, it also has immunosuppressive compounds, cyclosporine usually found in *Cordyceps subsessilis* Petch (Segelken, 1996). Besides this, some immunosuppressant compounds were also isolated from the closely related *Cordyceps* species *Isaria sinclairii* (Berk.) Lloyd (Mizuno, 1999).

**TABLE 1:** Chemical compounds of *Cordyceps* spp. (mentioned here only those compounds which were tested in a laboratory) adapted from Olatunji et al. with permission (Originally some portion of Table 3) (Olatunji et al., 2018).

Compounds	Species	Mode of action	References
Palmitic acid	*C. militaris* (L.) Fr.	Inactive	Yoon et al. (2015)
Linoleic acid			
Linoleic acid methyl ester			
Cordytropolone	*Cordyceps* spp. BCC 1681	Antimalarial, cytotoxicity against KB, BC-1, and Vero cells lines	Seephonkai et al. (2001)
Helvolic acid	*C. taii* Z.Q. Liang and A.Y. Liu	Cytotoxicity against HeLa, HepG2, 95-D and SW1990 cell lines	Dou et al. (2013)
Cordycepiamide A	*C. ninchukispora* C.H. Su and H.H. Wang) G.H. Sung, J.M. Sung, Hywel-Jones and Spatafora	Anti-inflammatory	Reis et al. (2013)
Cordycepiamide B			
Cordycepiamide C			
Cordycepiamide D			
*N*-(2-hydroxybenzyl)acetamide			
(-)-Syringaresinol			
Cordycerebroside A	*C. militaris* (L.) Fr*.*	Anti-inflammatory	Chiu et al. (2016b)
Soyacerebroside I			
Glucocerebroside			
Ergosterol peroxide	*C. cicadae* S.Z. Shing	Anti-inflammatory, renoprotective	Kuo et al. (2003)
Ergosta-4,6,8 (14),22-tetraen-3-one	*C. sinensis* (Berk.) Sacc.	Antitumor	Bok et al. (1999)
Jiangxienone	*C. jiangxiensis* Z.Q. Liang, A.Y. Liu & Yong C. Jiang	Cytotoxicity against SGC-7901 and A549 cell lines	Zhan et al. (2005)
H1-A	*C. sinensis* (Berk.) Sacc.	Renoprotective	Yang et al. (2003)
Cordyheptapeptide A	*Cordyceps* spp. BCC 16173	Cytotoxicity against KB, BC, NCI-H187, and Vero cells lines	Isaka et al. (2007b)
Cordyheptapeptide B	*Cordyceps* spp. BCC 16176	Cytotoxicity against KB, BC, NCI-H187, and Vero cells lines	
Cordycommunin	*Ophiocordyceps Communis* Hywel-Jones and Samson	Anti-mycobacterial	Haritakun et al. (2010)
Beauvericin J	*C. cicadae S.Z. Shing*	Cytotoxicity against HepG2 and HepG2/ADM cell lines	Wang et al. (2004)
Beauvericin			
Beauvericin A			
Beauvericin B			
Beauvericin E			
Cordyceamide A	*C. sinensis* (Berk.) Sacc.	Cytotoxicity against L929, A375, and Hela cell lines	Jia et al. (2009)
Cordyceamide B			
Cycloaspeptide A		Cytotoxicity against HeLa and MCF-7 cell lines	Zhang et al. (2009)
Cycloaspeptide C			
Cycloaspeptide F			
Cycloaspeptide G			
Cordycepin		Neuroprotection, anti-metastatic, anti-platelet aggregation, anti-inflammatory activity, anti-cancer	Yu H M et al. (2006)
N^6^-hydroxyethyl-adenosine	*C. pruinosa* Petch	Anti-inflammatory, Ca^2+^ antagonistic	Meng et al. (2015)
Guanosine	*C. sinensis* (Berk.) Sacc.	Immunomodulatory	Yu L et al. (2006)
CordysininB		Anti-inflammatory	Yang et al. (2011)
Dimethylguanosine		Antioxidant and HIV-1 protease	Jiang et al. (2011)
Ergosterol		Anti-inflammatory, anti-fibrotic	Nallathamby et al. (2015)
Ergosteryl-3-O-β-_D_-glucopyranoside		Anti-inflammatory, antioxidant	Bok et al. (1999)
5α,8α-epidioxy-22E-ergosta-6,9-(11)-22-trien-33β-ol		Cytotoxic against HL-60 cell line	Matsuda et al. (2009)
5α,6α-epoxy-5α-ergosta-7,22-dien-3β-ol			
5α,8α-epidioxy-24(R)-methylcholesta-6,22-dien-3β-D- glucopyranoside		Antitumor	Bok et al. (1999)
Cardinalisamide A	*C. cardinalis* G.H. Sung & Spatafora	Antitrypanosomal	Umeyama et al. (2014)
Cardinalisamide B			
Cardinalisamide C			
Cicadapeptin I	*C. heteropoda* Kobayasi	Antibacterial and antifungal	Krasnoff et al. (2005)
Cicadapeptin II			
Cordycepsidone A	*C. dipterigena* Berk. and Broome	Antifungal	Varughese et al. (2012)
Cordycepsidone B			
Cyclo (L-Pro-L-Val)	*C. sinensis* (Berk.) Sacc.	Antioxidant, anti-inflammatory	Yang et al. (2011)
Cyclo (L-Phe-L-Pro)			
Cyclo (L-Pro-L-Tyr)			
Cordycepoid A	*C. bifusispora* O.E. Erikss.	Inactive	Lu et al. (2013)
Cordysinin A	*C. sinensis* (Berk.) Sacc.	Anti-inflammatory, antioxidant	Yang et al. (2011)
Flazin			
Perlolyrine			
Cordyformamide	*C. brunnearubra* BCC 1395	Antimalarial	Isaka et al. (2007a)
Deacetylcytochalasin C	*C. taii* Z.Q. Liang and A.Y. Liu	Cytotoxicity against 95-D, A-549, and HL-7702 cell lines	Li et al. (2015)
Zygosporin D		Cytotoxicity against 95-D, A-549, and HL-7702 cell lines	
Cordypyridone A	*C. nipponica* Kobayasi	Antimalarial	Isaka et al. (2001)
Cordypyridone B			
Cordypyridone C			
Cordypyridone D			
1-Dehydroxycordypyridone A			
3′,4′,7-Trihydroxyisoflavone	*C. sinensis* (Berk.) Sacc.	Antioxidant	Yang et al. (2011)
Diadzein		Antioxidant, anti-inflammatory	
Rugulosin	*C. formosana* Kobayasi & Shimizu	Cytotoxicity against CHO cell line	Lu et al. (2014)
Skyrin			
6′-*O*-desmethylES-242–4	*Cordyceps* spp. BCC 16173	Antimalarial	Isaka et al. (2007b)
Annullatin A	*C. annullata* Kobayasi & Shimizu	Cannabinoid receptors agonist	Asai et al. (2012)
Annullatin B			
Annullatin C			
Annullatin D			
Annullatin E			
Erythrostominone	*C. unilateralis* (Tul.) Sacc.	Antimalarial, cytotoxicity against BC, KB, and Vero cell lines	Kittakoop et al. (1999)
Deoxyerythrostominone			
4-*O*-methyl-erythrostominone			
Epierythrostominol			
Deoxyerythrostominol			
Cordycepol	*C. ophioglossoides* (*T. ophioglossoides*) (Ehrh.) link	Cytotoxicity against HeLa and HepG2 cell lines	Sun et al. (2013)
Cordycepol B		Inactive	
Cordycepol C		Cytotoxicity against HeLa and HepG2 cell lines	
Cordycol			
Ophicordin	*C. sinensis* (Berk.) Sacc.	Antifungal	Kneifel et al. (1977)
Terreusinone A	*C. gracilioides* Kobayasi	Protein tyrosine phosphatases inhibitor	Wei et al. (2015)
Pinophilin C			
CryptosporioptideA			
Furancarboxylicacid	*C. sinensis* (Berk.) Sacc.	Anti-inflammatory, antioxidant	Yang et al. (2011)
Hydroxy-2-methyl-4-pyrone			
Bassiamide A (KTH-7-1)	*C. bassiana* Z.Z. Li, C.R. Li, B. Huang and M.Z. Fan	Antiproliferative against C6 glioma cell	Kim J H et al. (2015)
Bassiamate (KTH-7-2)			
IPr-PEPhenol (KTH-13)			
KTH-15–2			
KTH-17			
4-Quinolinol		Anti-inflammatory	Kim T W et al. (2014)
1-Naphthol			

### Cordycepin and Cordycepic Acid

Cordycepin and CA are prominently found in *C. militaris* (L.) Fr*.* They are important bioactive molecules having potential therapeutic applications (Huang et al., 2003). Structurally, cordycepin is 3′-deoxyadenosine and CA is D-mannitol. Cordycepin is an analog of adenosine derivatives which themselves differentiated from adenosine nucleoside by the absence of one oxygen molecule at third position carbon of ribose sugar. Different types of extraction methods of this compound can be followed, however, one of the most frequently used methods in which acetonitrile and water mixed in the ratio of 5:95 v/v at a flow rate of 1.0 ml/min can be adapted (Ikeda et al., 2008). Cordycepin has been associated with various therapeutic ailments including intracellular targets, nucleic acid, apoptosis, and cell cycle. This diverse role of cordycepins in cellular molecular activities is due to its resemblance to adenosine (Tuli et al., 2013b). On the other hand, CA is structurally an isomer of quinic acid possessing various potential medicinal applications. Previously, CA structure was concluded as 1,3,4,5-tetrahydroxycyclohexane-1-carboxylic acid (Chatterjee et al., 1957) later, it was found to be crystalline substance of D-mannitol (Sprecher and Sprinson, 1963). It differs mainly from quinic acid as it forms dextrorotatory instead of forming lactone (Chatterjee et al., 1957). There is a great variation of CA content in the *Cordyceps* spp. However, in *C. sinensis*, it is usually 7–29% with differing in growing stages of the *Cordyceps* spp. (Jiang, 1987). CA plays a great influence in treating liver fibrosis (Guo and Friedman, 2007), diuretic, plasma osmotic pressure, and anti-free radical properties (Nomani et al., 2014).

### Polysaccharides

*Cordyceps* spp. contains different types of polysaccharide components. The fruiting bodies of *Cordyceps* spp*.* consist of 3–8% polysaccharides (Li et al., 2001a). It was known that the polysaccharides obtained from *Cordyceps* species are medicinally important and can play as one of the main constituents in drug formulation (Ukai et al., 1983; Wasser, 2002). These polysaccharides can effectively control the blood sugar level in the body (Kiho et al., 1993), show antimetastatic and antitumor effects (Nakamura et al., 1999), and also have anti-influenza, immunoprotective, and antioxidant effects. *Cordyceps* spp. polysaccharides represent structurally diverse biologically active macromolecules of wide physiochemical properties. These polysaccharides are either intracellular or extracellular. Molecular weight greater than 16,000 is shown to have effective antitumor properties (Zhou et al., 2009). The polysaccharides derived from edible, medicinal mushrooms were successfully shown to exhibit antitumor and immunomodulating properties which were firstly reported from the fruit body of *Lentinusedodes* in 1969 (Chihara et al., 1969). Therefore, a large number of edible and medicinal polysaccharides including *Cordyceps* spp. have been rigorously investigated over the past 30 years. Apart from this, many novel antitumor and immunomodulatory polysaccharides have been developed and commercialized (Wasser, 2002; Xiao et al., 2002; Xiao et al., 2003). The important species of *Cordyceps* spp*.* from which polysaccharides have been isolated and developed which possess antitumor activities includes *C. sinensis*, *C. cicadae S.Z. Shing, C. ophioglossioides* (*Tolypocladium ophioglossoides* (Ehrh.) Quandt, Kepler & Spatafora)*, C. militaris* (L.) Fr. and *C. kyushuensis* A. Kawam*.* As per the study, the polysaccharides combined with other chemotherapeutic drugs showed synergism and increased body-resistance (Xiao et al., 2002; Yang et al., 2005; Zhang W et al., 2005; Chen et al., 2006). Polysaccharides derived from *Cordyceps* spp. primarily include glucan, mannan, heteroglycan, and glycoprotein but only β-(1→3) glucan, galactosaminoglycan, and proteopolysaccharide from *C. cicadae* S.Z. Shing, *C. ophioglossioides* and *Cordyceps* spp. showed antitumor activity (Xiao et al., 2002; Xiao et al., 2003).

### Proteins and Nitrogenous Compounds

*Cordyceps* spp. contains all essential amino acids, proteins, peptides, polyamines. Additionally, the *Cordyceps* spp. contains several rare cyclic dipeptides, including cyclo-[Gly-Pro], cyclo-[Leu-Pro], cyclo-[Val-Pro], cyclo-[Ala-Leu], and cyclo-[Thr-Leu]. Significant quantities of polyamines were also detected, such as 1,3-diamino propane, cadaverine, spermidine, spermine, and putrescine (Mizuno, 1999; Mishra and Upadhyay, 2011). Other nitrogenous compound like putrescine and putrescine, ware also identified (Mizuno, 1999).

### Nucleotides/Nucleotide Derivatives

Besides the other components, *Cordyceps* spp. is rich in nucleotide and its derivatives. In *C. sinensis*, nucleosides are the main component contributing to therapeutic applications (Li et al., 2001b). Nucleosides such as adenine, adenosine, inosine, cytidine, cytosine, guanine, uridine, thymidine, uracil, hypoxanthine, and guanosine have been isolated from *C. sinensis*. Among the nucleotide components, guanosine has the highest content ratio than other components (Shaoping et al., 2001). There is a usual difference between the nature of nucleosides from that of normal and cultured *C. sinensis* (Li et al., 2001c). Many specific nucleosides that are not found elsewhere in nature can be found in the *Cordyceps* spp. which includes several distinct deoxyuridin structures, adenosine, 2′-3′-dideoxyadenosine, hydroxyethyladenosine, cordycepin triphosphate, guanidine, and deoxyguanidine. Adenosine and cordycepin (3′-deoxyadenosine) possess multiple functions such as immunomodulatory, antioxidant, etc., Chen and Chu (Chen and Chu, 1996), identified cordycepin by using magnetic resonance (NMR) and infrared spectroscopy (IR) in a *C. sinensis* sample. In identification of cordycepin, several analytical methods and techniques including RP-HPLC (Shiao et al., 1994; Yu H M et al., 2006; Yu L et al., 2006), HPLC–ESI-MS (Huang L F et al., 2004), and HPLC-DAD (Jiang et al., 2008) were adopted.

### Sterols and Fatty Acid

Fungi contain sterols in the form of ergosterol an essential part of the great therapeutic important part of vitamin D_2_. *Cordyceps* spp. has identified a host of several sterol-type compounds and a few of these names: ergosterol, ergosterol-3, ergosterol peroxide, 3-sitosterol, daucosterol, and campesterol (Zhou et al., 2009). In *Cordyceps* spp., the existence of ergosterol varies depending on their growth stage, i.e., ergosterol was 1.44 mg/g in *Cordyceps* spp. mycelium, while 10.68 mg/g in fruit bodies (Li et al., 2011). Some derivatives of *Cordyceps* spp. are found in D-3-ergosterol, 3-sitosterol, daucosterol, and campesterol, and so on. It is important to mention that HPLC in *C. sinensis* detects ergosterol (Li and Li, 1991; Li et al., 2004).

The fatty acids found in *Cordyceps* spp. can be classified loosely into two kinds of fatty acids, saturated and unsaturated. *Cordyceps* spp. are more common and can compensate for up to 57.84% of unsaturated fatty acids (Zhou et al., 2009). Fatty acid such as lauric acid, myrtic acid, pentadecanic acid, palmitic acid, linoleic acid, oleic acid, stearic acid, and docosanic acid, are reported in *Cordyceps* spp*.* (Mishra and Upadhyay, 2011). Zhu et al. (1998), reported that 28 saturated and unsaturated fatty acids and their derivatives were isolated from *C. sinensis* along with polar compounds include several alcohols and aldehydes. The unsaturated fatty acids have various physiological activities, including decreased lipid blood and cardiovascular disease. Two methanol isolated sterols displayed antitumor sequence, and were detected by 1D and 2D NMR spectroscopy in their structure (Bok et al., 1999). Usage of pressurized fluid extraction (PLE), derivation of trimethyl silyl (TMS), GC-MS, cholesterol, campesterol, and β-sitosterol, like ergosterol from natural (wild) *C. sinensis* were described (Yang et al., 2009).

### Other Constituents

In addition to the core ingredients, *C. sinensis* is made mostly from proteins, peptides, polyamine, both important amino acids, and other unusual cyclic dipeptides such as cyclo-[Gly-Pro-], cyclo-[Leu-Pro-], cyclo-[Val-Pro] and cyclo-[Thr-Leu]. Cyclic dipeptides including cyclo-(Leu-Pro) and cyclo-(Phe-Pro) were seen to have antimicrobial activity and anti-mutagenic properties in the battle against the production of vancomycin-resistant *Enterococcus* (VRE) and pathogenic yeasts (Rhee, 2004). As per the study, cyclic (bacterial) dipeptides inhibit the development of aflatoxin (Yan et al., 2004) and protein rates differ greatly in the sum of dead larvae (29.1%), fruit body (30.4%), and mycelial fermentation (14.8%). The major amino acids are present in the larvae such as glutamic acid, aspartic acid, and amino acid (Hsu et al., 2002). The anti-inflammatory and anti-nociceptive properties of cordymine, a peptide isolated from *C. sinensis* medicinal mushrooms, have been reported (Qian et al., 2012).

The exopolysaccharide fraction (EPSF), is derived from the harvested *C. sinensis* supernatant. The cultured supernatant has been collected and then processed with the three times in volume of 95% ethanol for precipitation. As a consequence, a large amount of EPSFs was found on the soil (Zhang et al., 2008). EPSF has a wide spectrum of pharmacologic effects, with immunomodulatory and antitumor effects are most important (Sheng et al., 2011). EPSF has already shown that it can scavenge free radicals, promote the differentiation of cell cancer, and improve the ability of antitumor activity by triggering many immune responses (Sheng et al., 2011). Ion-exchanging and size chromatography is used to isolate polysaccharide (PS) from cultivated *C. sinensis* mycelia. Polysaccharide fraction (PSF) has been extracted from *C. sinensis* fungus has a relaxing effect on macrophage (Chen W et al., 2010). PSF has been shown to transform M_2_ macrophages to M_1_ phenotypes by activating the nuclear factor kappa-B (NF-κB) pathway. PSF also has immunomodulatory impacts, including many other polysaccharides (Chen et al., 2012). In a study to document the effect of *C. sinensis* on T-lymphocyte subsets of chronic renal failure patients, it was reported that different components of *Cordyceps* spp. polysaccharides enhanced the cellular immune function, phagocytic function of monocyte-macrophage, improved renal functions, spleen, and thymus index (Guan et al., 1992).

## Extraction and Isolation of Major Compounds From *Cordyceps* spp.

### Extraction

A few extraction strategies have been used for solvents extraction utilized for the confinement of particular bio-dynamic mixes (Chen P X et al., 2013). Different extracts exhibit significant biological activities.

#### Aqueous Extraction

In aqueous extraction, water is used as an extraction medium due to the polar nature of the molecule and extracts polar compounds like-nucleosides and polysaccharides. Sun et al. (2003) standardized the suitable conditions for aqueous extraction as water: plant powder ratio (2.5:1), pH-7.5–8.0, and 24 h extraction time (Sun et al., 2003). Moreover, in hot water extraction, the yield varies between 25–30% with potential health benefits like antioxidant activities (Yamaguchi et al., 2000a; Gu et al., 2003).

#### Alcoholic Extraction

An alcoholic extraction method mainly methanol, ethanol, aqueous methanol, and aqueous ethanol are used for extraction as per bioactive principles. Yamaguchi et al. studied the alcoholic extraction because it allows a higher extraction of bioactive molecules, such as nucleosides, polysaccharides, proteins, as a result, exhibits strong antioxidant activity and preserves B-cell function and provides protection (Yamaguchi et al., 2000a; Kan et al., 2012). Another study revealed that methanol extract obtained from *C. sinensis* was found to have cytotoxicity impact on cancer cell lines (Jia et al., 2009).

#### Ethyl Acetate Extraction

Ethyl acetic acid derivation concentrate *C. sinensis* includes an intensification range not as similar to water and alcohol. Although the yield in this technique is small, the technique includes sugar, adenosine, ergosterol, and cordycepin, which are differentiated by ergosterol and similar mixes as a significant class of dynamic portion. The cause of apoptosis in human pre-myelocytic leukemia HL60 is due to 2 days of treatment in ED 50 ± 25 µg/ml, as a result, restrains the proliferation of malignancy growth of the cell lines (Zhang et al., 2004; Wu et al., 2007). Further research is utilized to comprehend basic highlights and adequacy of dynamic mixes in ethyl acetic acid derivation extricate. Ethyl acetate extract of *C. sinensis* showed antioxidant and immunomodulatory potential (Wu et al., 2006; Wu et al., 2007).

#### Supercritical Carbon Dioxide (CO_2_) Extraction

The extraction of supercritical CO_2_ has been an emerging technique in the chemical and food sectors in recent years. It is the best method carried out under moderate conditions and its purest form to extract bioactive compounds (especially non-polar compounds), without toxic organic solvents for extraction. Many literatures on simple and supercritical methods for the extraction of fluids in different fields are available (Pereira and Meireles, 2010). Ethanolic *C. sinensis* extract was fractionated with supercritical CO_2_ as an elution solvent, demonstrating its strong scavenging potential and inhibiting colorectal and hepatocellular cell development via the apoptosis cycle (Wang et al., 2005).

## Pharmacological Potential of *Cordyceps* spp.

Plethora of naturally occurring chemical entities attributes to the broad and remarkable pharmacological activities of *Cordyceps* spp*.* (Zhu et al., 1998; Tuli et al., 2013a). Out of the diverse variety of species, *C. sinensis* is the most investigated one, as far as research and the inspection of its pharmacological potential is concerned (Paterson, 2008; Olatunji et al., 2018). Besides, other species includes *C. militaris* (L.) Fr.; *C. pruinosa* Petch; *C. ophioglossoides (T. ophioglossoides*); *C. bassiana* Z.Z. Li, C.R. Li, B. Huang and M.Z. Fan; *C. guangdongensis* T.H. Li, Q.Y. Lin and B. Song; *C. gunnii* (Berk.) Berk*.*; *C. jiangxiensis* Z.Q. Liang, A.Y. Liu & Yong C. Jiang; *C. kyushuensis* A. Kawam.; *C. pseudomilitaris* Hywel-Jones and Sivichai; *C. sphecocephala* (Berk.) Sacc; *C. soblifera* (Hill ex Watson) and *C. taii* Z.Q. Liang and A.Y. Liu. The proposed applications of *Cordyceps* spp. in medicine include as immune-stimulatory, immunomodulatory, anti-inflammatory, antioxidant, antitumor, antimetastatic, antibacterial, antifungal, antimalarial, HIV-1 protease inhibitor, antihyperlipidemic, anti-obesity, anti-diabetic, anti-arteriosclerosis, anti-thrombotic, anticoagulant, anti-fatigue (Qian et al., 2012; Liu Y et al., 2015). Details of the *Cordyceps* spp. induced pharmacological actions have been described as below.

### Immuno-Modulatory Action of Cordyceps spp.

**FIGURE 2:**
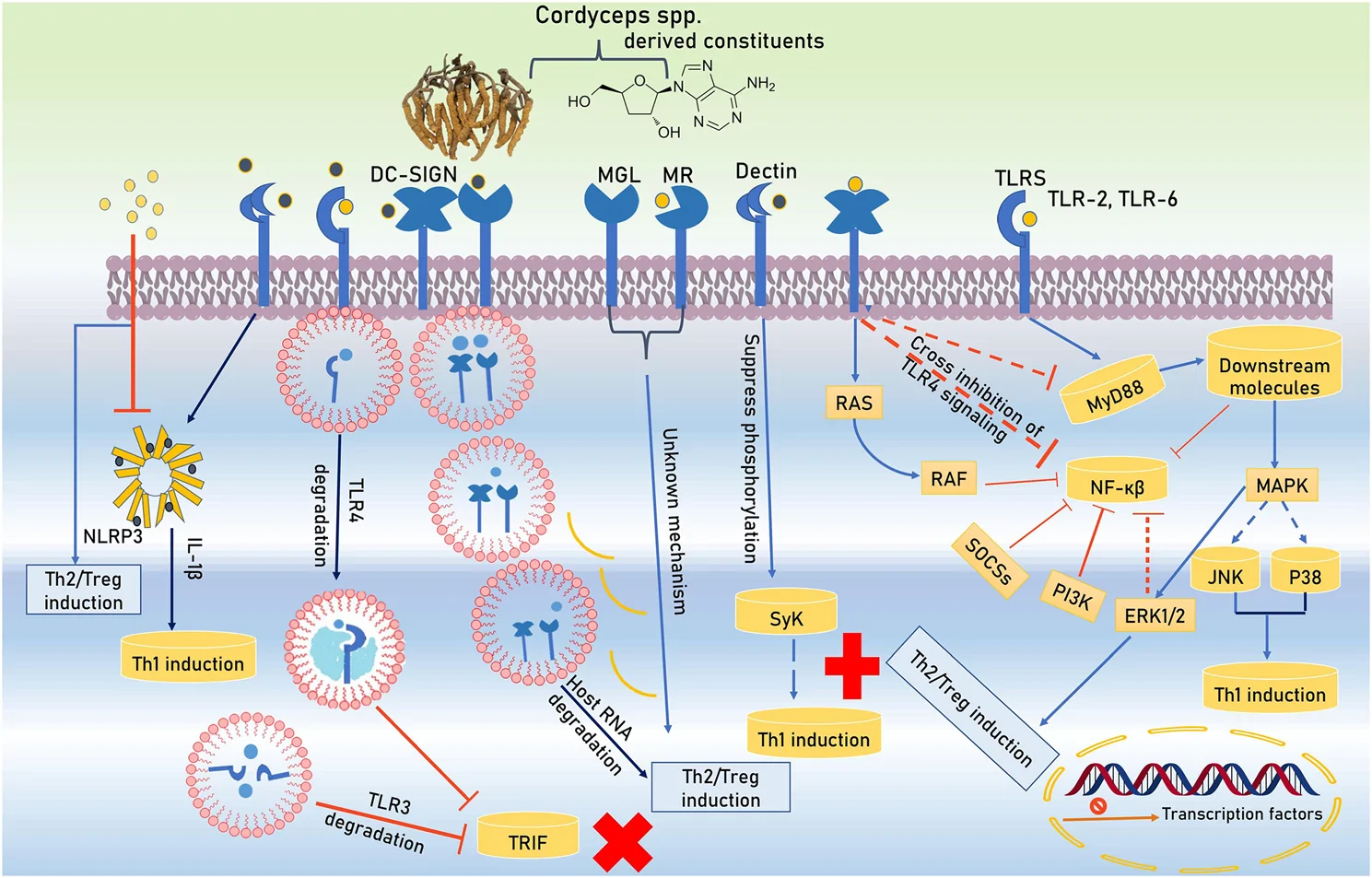
Signal pathway activation by TLRs and CLRs and its interaction with the *Cordyceps* spp. derived constitutes.

The immunomodulators are the substances or compounds that helps to control the immune system of the body. There are a number of compounds present in the *Cordyceps* spp. that possesses the immunomodulatory activity. Some of these are discussed below. Active constitutes of *Cordyceps* spp. are spotted by Toll-like receptors (TLRs) and C-type lectin receptors (CLRs) during initiation of immunomodulation and hyporesponsiveness in antigen-presenting cells (APCs). These active constituents not only alter the TLRs and CLRs expression in APCs but also masterfully manipulate their intracellular signaling. TLRs use the Toll/IL-1 receptor (TIR)-domain covering adapter proteins such as MyD88 and TRIF (TIR domain-containing adapter inducing IFN-β). Active bio-constituents of *Cordyceps* spp. (*C. cicadae* S.Z. Shing, *C. militaris* (L.) Fr., *C. sinensis*, *C. sobolifera* (Hill ex Watson)) transmit TLR4 signaling to MAPK pathway and extracellular signal-related kinase one and 2 (ERK1/2) activation backing Treg/Th2 induction. Furthermore, coherence of DC-SIGN (dendritic cell-specific intercellular adhesion molecule-3-grabbing non-integrin) along with TLR4 enables active constituents of *Cordyceps* spp. to trigger unknown intracellular pathways that cross-inhibit MyD88 and NF-κB activation. These constituents are further restrained NF-κB activity via the upregulation of negative regulators of TLRs signaling like a suppressor of cytokine signaling (SOCS) and phosphatidylinsoitol-3-kinase (PI3K) along with DC-SIGN-mediated rapidly accelerated fibrosarcoma (RAF) signaling. In the prevention of priming Th1 cells, the role of NF-κB is a core factor due to its support’s inflammation by inhibition. The multiplicity of signaling pathways is improved by co-receptors' involvement of CLRs (DC-SIGN). Activated mannose receptor (MR) and macrophage galactose-type C-type lectin (MGL) helps for the differentiation of Treg/Th2. Degrading host key intracellular molecules is another strategy that *Cordyceps* spp. exploit to reprogram host immunity. Polysaccharide constituents of *Cordyceps* spp. degrades endosomal TLR2, TLR3, TLR4, TLR6, and host mRNA which provides Treg/Th2 responses support. The active bio-constituents stimulate Treg/Th2 cell priming which have been stated by CLRs involvements. NLRP3 inflammasome (NLRP3 and caspase-1) modulate inflammatory processes via secretion of IL-1β and Th1 intensification (Figure 2).

Xu et al. (1992) delineated the effects of the *C. sinensis* (ethanolic extract) on murine and human natural killer (NK) activity and on colony formation of B16 melanoma in mouse lungs, where they reported the augmentation of the *in vivo* and *in vitro* NK activities of the mouse. Moreover, the pre-incubation of peripheral blood mononuclear cells (PBMCs) with *C. sinensis* elevated *in vitro* NK activity of human PBMCs, whereas the colony formation of B16 melanoma in mouse lungs was reduced drastically. This report hinted at the *C. sinensis* immunopotentiation in immunodeficient patients (Xu et al., 1992). Interestingly, the induction of macrophages and the intestinal immune system in mice by oral administration of hot water decoction from cultured mycelia of *C. sinensis* has also been reported (Koh et al., 2002). They inferred modulatory IL-6 production by activating macrophages and enhance secretion of hematopoietic growth factors like granulocyte-macrophage colony-stimulating factor (GM-CSF) and IL-6 from Peyer's patch cells (mainly composed of T and B cells) (Koh et al., 2002). *Cordyceps* spp. induced modulation of cytokines has been reported by others as well (Yu et al., 2004). *C. sinensis* play an immunomodulatory role in the pathogenesis of GAS (Group A *Streptococcus*) infection in U937 cells by inducing the expression of cytokines like IFN-γ, IL-12, and TNF-α, that eventually augmented the phagocytosis (Kuo et al., 2007). *C. militaris* (L.) Fr. polysaccharides (CMP) induced immune activation were studied in cyclophosphamide-induced immunosuppressed mice by assessing the lymphocyte proliferation, phagocytic index, and other biochemical parameters (Wang M et al., 2012), thus hinting its use as a future immunomodulatory agent.

The immuno-stimulatory action of a compound is explicated by its competence to trigger the immune system of the living organism through inducing or activating its components. Numerous species of *Cordyceps* spp. exhibits immuno-stimulatory activities in distinct parts of the body (Table 2). The use of *C. sinensis* has been documented in the medicament of respiratory infections by activating the immune response via innate immunity promotion (Lin and Li, 2011). *Cordyceps* spp. also promotes the adaptive immune system, comprising the cellular and humoral immunity (Lin and Li, 2011). Zhu et al. (2012b) investigated the role of *C. gunnii* (Berk.) Berk*.*-derived polysaccharides for immunostimulatory and antitumor purposes, and cytokines expression in normal, immuno-compromized, and H22-bearing mice. They inferred that the polysaccharides from the *C. gunnii* (Berk.) Berk*.* probably boost the non-specific immunity, humoral and cellular immunity, and restrain the tumor growth. CP2-S, (a novel polysaccharide) purified from *C. militaris* (L.) Fr. exhibits immunostimulatory activity by inducing phagocytosis, NO production, respiratory burst, and secretion of IL-1β and IL-2 (from macrophages). Bi et al. (2018) reported the immunostimulatory action of the novel polysaccharide (low-molecular-weight) obtained from the fruiting bodies (cultured) of *C. militaris* (L.) Fr. in splenic lymphocytes and natural killer cells through induction of MAPK, NF-κB, and Toll-like receptor (TLR) two pathways. Ethanol extracts of *C. sinensis* enhance phagocytosis activity as evidenced by carbon clearance in tumor-bearing mice. It also caused a remarkable increment in an acid phosphatase activity and lysosomal enzymes in macrophages suggesting its antitumor action via the immuno-stimulating function (Shin et al., 2001; Shin et al., 2003).

**TABLE 2:** Immunostimulatory and related bioactivities of *Cordyceps* spp.: *in vitro* and *in vivo*.

Activity	Species	Extract/Compound	*In vitro/in vivo*	Results	References
Immunostimulant	*C. militaris* (L.) Fr.	Polysaccharides	RAW264.7 macrophages	↓NO, ROS, TNF-α production, NF-κB activation and MAPKs pathways, melanoma growth	Lee and Hong (2011)
				↓NO, TNF-α and IL-1β production	Lee et al. (2010)
				↓NO, TNF-α and activates macrophages through the MAPKs and NF-κB signaling pathways	Lee et al. (2015)
	*C. japonica* Lloyd	Aqueous and methanol	Forced swimming performances, immobilizing stress	↑Liver enzyme activities, ↓lipid peroxidation	Shin et al. (2001)
Immunomodulatory	C. bassiana Z.Z. Li, C.R. Li, B. Huang and M.Z. Fan Z.Z. Li, C.R. Li, B. Huang and M.Z. Fan	Ethanol	LPS-activated macrophages	↓Expression of IL-12, IFN-γ	Byeon et al. (2011a)
		Butanol fraction	Molecular basis of inhibition of cytokine expression	↓IL-12 and TNF-α expression, Syk, JAK-2, and ERK	Byeon et al. (2011b)
		Methanol	HMNC proliferation	↓HMNC proliferation, EC_50_ = 32.5 ± 5.2 μg/ml, ↑IL-2 and IFN-γ	Weng et al. (2002)
	*C. gunnii* (Berk.) Berk.	Polysaccharide	Macrophage phagocytosis along with humoral and cellular immunity	↑Thymus and spleen indexes, macrophage phagocytosis, the proliferation of splenic cells, level of IFN-γ and TNF-α, ↓IL-4	Zhu et al. (2012b)
			Spleen lymphocytes proliferation, peritoneal macrophage (PMphi) phagocytosis, and CTL	↓Spleen lymphocytes proliferation, PMphi phagocytosis of neutral red and CTL	Xiao et al. (2004b)
	*C. pruinosa* Petch	Polysaccharide	Splenic T cell and Mphi phagocytosis	↑Proliferation of activated splenic T cell and cellular immune functions	Liu and Fei (2001)
			Renal injury in endotoxemic rats	↓Oxidative stress and inflammatory cytokines, NF-κB activation, ↑body’s cellular antioxidant defense system.	(Chiu et al., 2014)
	*C. taii* Z.Q. Liang and A.Y. Liu	Polysaccharides	Splenic T cell and Mphi phagocytosis	↑Proliferation of activated splenic T cell and cellular immune functions	(Liu and Fei, 2001)
	*C. sinensis* (Berk.) Sacc.	Adenosine and guanosine	RAW264.7 cells	↓NO, ↑IL-1β, TNF-α	(Yu et al., 2007)
		Polysaccharide	Macrophages proliferation, phagocytosis	Activation of the MAPK and NF-κB signaling pathways	(Cheong et al., 2016)
		Exopolysaccharide	B16 melanoma-bearing mice	↑Neutral red uptake capacity, spleen lymphocyte proliferation, ↓levels of Bcl-2	Zhang Q et al. (2005)
		Aqueous	Lupus-prone (NZB/NZW) F1 hybrids	↑Survival, CD8^+^ T cells%, ↓ proteinuria, titers of anti-double-stranded DNA antibody CD4^+^ T cells%	(Chen et al., 2009)
			Macrophage J774 cell	↑Phagocytosis	(Jia and Lau, 1997)
		Exopolysaccharide	Raw264.7 macrophage cell	Stimulate the release of cytokines	(Wang et al., 2011)
		Chloroquine and bafilomycin A	Bone marrow-derived dendritic cells (BM-DCs)	Activation of BM-DCs in a TLR9-dependent manner	(Xiao et al., 2010)
		Methanol	Lymphoproliferative response, natural killer cell (NK)	Inhibited blastogenesis response, NK cell activity, IL-2, and TNF-α production	(Kuo et al., 1996)
Immunomodulatory and antioxidant	*C. militaris* (L.) Fr.	Polysaccharides	Cyclophosphamide-induced immunosuppression	↑Spleen lymphocyte activity, macrophage function, SOD, catalase, GPx, and TAOC level and the spleen and thymus indices, ↓MDA level	Wang M et al. (2012)
		Polysaccharides	Viscera index, leukocyte count, differential leukocyte count, IgG levels	Upregulated the expression of TNF-α, IFN-γ, and IL-1β mRNA, ↑spleen and thymus indices, the spleen lymphocyte activity, the total quantity of white blood cells, and IgG function, ↓MDA	(Liu et al., 2016)
Immunosuppressive	*C. gunnii* (Berk.) Berk.	Polysaccharide	Cytotoxic T lymphocytes	Inhibiting cellular immunologic and humoral immunologic function	(Xiao et al., 2004a)
Anti-inflammatory	C. bassiana Z.Z. Li, C.R. Li, B. Huang and M.Z. Fan	1,9-Dimethylguanine	Reporter gene assay and mRNA analysis	Blockade of luciferase activity caused by NF-κB and AP-1, suppress the mRNA levels of COX-2 and TNF-α	(Suh et al., 2017)
		Butanol fraction	LPS-induced inflammation in RAW 264.7 cells	↓NO, iNOS, COX-2, IκB, MAPKs activation, JNK, and p38 phosphorylation	(Yoon et al., 2017)
		4-Isopropyl-2,6-bis(1-phenylethyl) phenol	LPS and sodium nitroprusside treated RAW264.7 cells	↓NO and ROS production, mRNA expression, NF-κB activation	(Yang et al., 2015b)
		Aqueous	LPS-treated RAW264.7 cells.	↓COX-2, IL-12, and iNOS, Syk kinase activity ↑IL-10	(Yang et al., 2017)
		Butanol fraction		↓NO and ROS production, and IκB/NF-κB pathway, JNK, and p38 activation	Kim T W et al. (2014)
	*C. cicadae* S.Z. Shing	Ergosterol peroxide	Human T cells	↓T-cell proliferation, IL-2, IL-4, IL-10, and IFN-γ, AP-1 proteins expression	(Kuo et al., 2003)
		N^6^-(2-Hydroxyethyl) adenosine	LPS-induced pro-inflammatory	↓TLR4-mediated NF-κB signaling pathway	(Lu et al., 2015)
			CCl_4_-induced liver fibrosis	↓BUN and SCr levels, IL-12 and TNF-α expression, TGF-β1/CTGF	(Kim et al., 2018)
	*C. guangdongensis* T.H. Li, Q.Y. Lin and B. Song	Aqueous	Chronic bronchitis caused by tobacco smoking	↓Bronchial lesions and inflammatory cell infiltration	(Yan et al., 2014)
	*C. militaris* (L.) Fr.	Aqueous	Dextran sodium sulfate-induced acute colitis	Attenuated body weight loss, diarrhea, gross bleeding, ↓epithelial damage, loss of goblet cells, loss of crypts, infiltration of inflammatory cells	(Han et al., 2011)
		Cordycerebroside A soyacerebroside I and glucocerebroside	RAW264.7 macrophages	↓Accumulation of pro-inflammatory iNOS and COX-2 protein expression	(Chiu et al., 2016b)
	*C. pruinosa* Petch		RAW264.7 macrophage cells	↓ NO production, TNF-α, ROS, IL-6, iNOS, phosphorylation of p65/p50	Kim H G et al. (2014)
		Methanol		↓IL-1β, TNF-α, COX-2, iNOS, NF-κB activation	(Kim et al., 2003)
Anti-inflammatory and anti-cancer	*C. militaris* (L.) Fr.	Cordycepin	LPS/IFN-γ-stimulated macrophages and colon 205, PC-3, and HepG2 cells	↓NO, TNF-α and IL-12 production, IC_50_ = 7.5, 6.3, and 7.6 μg/ml	(Rao et al., 2010)
Antioxidant	*C. cicadae* S.Z. Shing	Water-soluble polysaccharides	Total reducing power and scavenging activities	IC_50=_ 28.99 μg/ml (DPPH scavenging), 0.19 and 0.30 mg/m L mg/ml (hydroxyl and superoxide anion radicals)	(Song et al., 2018)
	*C. formosana* Kobayasi & Shimizu	Aqueous	DPPH and ROS scavenging	Strong antioxidant capability	Wang Y W et al. (2015)
	*C. gunnii* (Berk.) Berk.	Polysaccharide	D-galactose-induced	↓LPO, GPx, ↑SOD, catalase	(Zhu et al., 2009)
				↓MDA, GPx, ↑SOD	(Zhu et al., 2011)
	*C. japonica* Lloyd	Aqueous and methanol	TBA reactant assay	↑Cytosolic SOD, catalase, and GSH-px, ↓MDA	(Shin et al., 2001)
		Ethanol	DPPH	IC_50_ = 163 μg/ml, ↑SOD, catalase	(Jung et al., 2009)
	*C. jiangxiensis* Z.Q. Liang, A.Y. Liu & Yong C. Jiang	Polysaccharide	DPPH	EC_50_ = 18.06 mg/ml	(Xiao et al., 2011)
	*C. kyushuensis* A. Kawam.	Methanol	Scavenging effect on hydroxyl radical	IC_50_ = 1.5–4.8 mg/ml	(Zhang et al., 2015)
			
	*C. militaris* (L.) Fr.	Polysaccharides	DPPH, hydroxyl and superoxide radical	Strong antioxidant capability	Chen X et al. (2013)
		Methanol		↓LPO also scavenge reducing power and free radicals.	(Reis et al., 2013)
	*C. pruinosa* Petch	Polysaccharide	DPPH, hydroxyl, and superoxide radical	↓LPO also scavenge reducing power and free radicals.	(Lu et al., 2016)
	*C. sinensis* (Berk.) Sacc.	Polysaccharide	PC12 cells against hydrogen peroxide-induced injury	↑SOD, GSH, ↓MDA	(Gu et al., 2003)
		Aqueous and ethanol	DPPH, hydroxyl, and superoxide radical	↓LPO, scavenge reducing power and free radicals	(Yamaguchi et al., 2000a)
		Exopolysaccharide	Trolox equivalent antioxidant	35–40 μmol Trolox/g	(Leung et al., 2009)
Antioxidant and Immunoenhancing	*C. taii* Z.Q. Liang and A.Y. Liu	Polysaccharides	D-galactose-induced aging	Superoxide anion-free radical (EC_50_ = 2.04–2.49 mg/ml), ↑ SOD, catalase, GSH, ↓MDA	(Xiao et al., 2012b)
Free radical scavenging	C. bassiana Z.Z. Li, C.R. Li, B. Huang and M.Z. Fan	Methanol	DPPH radical inhibition	47.7% scavenging activity of stage 3	(Hyun et al., 2013)
Anti-tumor	*C. sinensis* (Berk.) Sacc.	Methanol	K562, Jurkat, WM-1341, HL-60, and RPMI-8226 cells	10–40% at 10 μg/ml inhibitor to the proliferation	(Bok et al., 1999)
		Polysaccharide	U937 cells	78–83% growth inhibition rate, ↑IFN-γ, and TNF-α	(Chen et al., 1997)
		Cordycepin	K562, Vero, Wish, Calu-1, and Raji tumor cell lines	Significantly inhibited	(Kuo et al., 1994)
	*C. japonica* Lloyd	Ethanol	Sarcoma-180 tumor cells	↑Phagocytosis and acid phosphatase activity	(Shin et al., 2003)
	*C. gunnii* (Berk.) Berk.	Polysaccharide	S180 cells	Stronger inhibition at 800 μg/ml	(Zhu et al., 2016a)
			K562 cell	56.65% tumor inhibition ratio	(Zhu et al., 2012a)
			H22 cell	45.3% tumor inhibition ratio	Zhu Z-Y et al. (2014)
			K562 cells	69.92% tumor inhibition ratio	(Zhu et al., 2013)
		Selenium enriched polysaccharide	SKOV-3 cells	Stimulate apoptosis through p53-Bax-caspase pathway	(Sun et al., 2018)
		Polysaccharide	S180 cell	85% tumor inhibition ratio	(Zhu et al., 2016b)
	*C. cicadae* S.Z. Shing	Ethanol	SGC-7901 cells	↓Proliferation of SGC-7901 cells, ↑calpain-1, caspase-12, and caspase-9 expression	(Xie et al., 2019)
Anti-tumor and antimetastatic	*C. taii* Z.Q. Liang and A.Y. Liu	Chloroform	A549 and SGC-7901 cells	IC_50_ = 30.2 and 65.7 μg/ml, ↑GPx	Liu R M et al. (2015)
Anti-cancer	*C. cicadae* S.Z. Shing	Aqueous	MHCC97H human hepatocellular carcinoma cells	↓MHCC97H cells growth via G2/M cell cycle arrest	Wang H et al. (2014)
	*C. formosana* Kobayasi & Shimizu	Aqueous	A549 lung cancer, MDA-mb-231 breast cancer, Huh7 liver cancer, and HL-60 leukemia cells	IC_50_ = 1.0 mg/ml (A549 cells), IC_50_ = 0.53 mg/ml (MDA-mb-231 cells), IC_50_ = 0.44 mg/ml (Huh7 cells), IC_50_ = 0.19 mg/ml (HL-60 cells), ↓breast tumor size	Wang J et al. (2014)
	*C. kyushuensis* A. Kawam.		U937 and K562 cells	IC_50_ = 31.23 μg/ml and 62.5 μg/ml	(Zhao et al., 2018)
	*C. jiangxiensis* Z.Q. Liang, A.Y. Liu & Yong C. Jiang	Chloroform	Gastric adenocarcinoma cell line SGC-7901	IC_50_ = 10 μg/ml, ↑ caspase-3 activity	(Xiao et al., 2006)
	*C. militaris* (L.) Fr.	Curdlan	Dendritic cell maturation	↑ CD40, CD80, CD86, MHC-I, MHC-II molecules, IL-12, IL-1β, TNF-α, IFN-αβ expression, phosphorylation of ERK, p38, JNK, and NF-κB, p50/p65	Kim H S et al. (2010)
	*C. taii* Z.Q. Liang and A.Y. Liu	Cytochalasin	95-D, A-549 and HL-7702 cells	IC_50_ = 3.67–4.04 μM	(Li et al., 2015)
	*C. sphecocephala* (Klotzsch ex Berk.) Berk. and M.A. Curtis	Polysaccharides	HepG2, SKN-SH cells	Activation of caspase-3, and modulation of Bcl-2 and Bax	(Oh et al., 2008)
Cytotoxicity	*C. bifusispora* O.E. Erikss.	Methanol	CHO cells	Cell death ratio (4.14 ± 0.25) at 4,000 μg/ml concentration, LD_50_ > 8.0 g/kg	(Lu et al., 2013)
	*C. cicadae* Shing.	Beauvericin, beauvericin A, beauvericin E, and beauvericin J	HepG2 and HepG2/ADM cells	IC_50_ = 2.40 ± 0.37 to 14.48 ± 1.68 μM.	Wang J et al. (2014)
	*C. formosana* Kobayasi & Shimizu	Rugulosin and skyrin	CHO cells	Rugulosin and skyrin LD_50_ = 18.3 ± 0.2 and 103.7 ± 5.9 μg/ml	(Lu et al., 2014)
	*C. jiangxiensis* Z.Q. Liang, A.Y. Liu & Yong C. Jiang	Jiangxienone	SGC-7901 cell and A549 cell	IC_50_ = 1.38–2.93 μM	(Xiao et al., 2012a)
			HGC-27	DNA damage response pathway	(Lü et al., 2014)
	*C. pruinosa* Petch	Cordycepol C, cordycol	HeLa and HepG2	IC_50_ = 12–33 μg/ml	(Sun et al., 2013)
		Butanol fraction	HeLa	Caspase-3- and -9-dependent apoptosis, ↑proteolytic cleavage of PARP and release of cytochrome c, ↓Bcl-2/Bax protein ratio	Kim H G et al. (2010)
Anti-diabetic	*C. cicadae* S.Z. Shing	Crude polysaccharide	Alloxan-induced diabetic	↓Blood glucose, TC, TG, LDL, MDA, urea, CREA, ALT, AST, and ALP. ↑body weights, HDL, SOD, GSH	(Zhang et al., 2018)
	*C. japonica* Lloyd	Methanol	STZ-induced diabetes	↓serum glucose, glucose tolerance up to 3 h	(Shim et al., 2000)
	*C. militaris* (L.) Fr.	Cordycepin	Alloxan-induced	↓Blood glucose, TC, TG, LDL, MDA, urea, CREA, ALT, AST, and ALP. ↑body weights, HDL, SOD GSH	(Ma et al., 2015)
Antimicrobial	*C. cicadae* S.Z. Shing	Hydroalcoholic	Agar well diffusion method	Damage bacterial cell wall and membranes, ↑cell permeability	(Zhang et al., 2017)
Antibacterial	*C. heteropoda* Kobayasi	Cicadapeptins I and II	Agar disk/diffusion assays	Inhibition zones against *Bacillus cereus* (13 and 12 mm) and *B. subtilis* (13 and 11 mm), *Escherichia coli* (16 mm for both peptides)	(Krasnoff et al., 2005)
Antifungal		Cicadapeptins I and II	Potato dextrose agar plates	Botrytis cinereal (11 mm zones) showed inhibitory activity	(Krasnoff et al., 2005)
	*C. dipterigena* Berk. and Broome	Cordycepsidone A	Gibberella fujikuroi	Strong and dose-dependent activity	(Varughese et al., 2012)
Neuroprotective	*C. cicadae* S.Z. Shing	Polysaccharides, adenosine	Glutamate-induced PC12 cells	↑Cell survival rate, GPx, SOD, Bcl-2/Bax ratio, ↓ROS and Ca^2+^, ERK, p38, and JNK expression	(Olatunji et al., 2016)
		Butanol fraction	Glutamate-induced PC12 cells	↓ROS accumulation, GSH-Px, and SOD levels	(Wang et al., 2018)
Antiviral	*C. guangdongensis* T.H. Li, Q.Y. Lin and B. Song	Aqueous	Influenza virus H9N2	↓Pulmonary index by 22.1%	(Yan et al., 2010)
Antimalarial	*C. brunnearubra* BCC 1395	Ethyl acetate	Malarial parasite plasmodium falciparum K1	IC_50_ = 18 μM	(Isaka et al., 2007a)
Cannabinoid receptors CB1 and CB2 (agonistic)	*C. annullata* Kobayasi & Shimizu	Ethyl acetate	HEK293 cells	15.5–75.5% inhibition	(Asai et al., 2012)
Anti-proliferative	C. bassiana Z.Z. Li, C.R. Li, B. Huang and M.Z. Fan	Ethanol	VSMC and carotid artery of balloon-injured rats	↓VSMC proliferation and ↑ERK 1/2 phosphorylation	(Jin et al., 2016)
Anti-trypanosomal	*C. cardinalis* G.H. Sung & Spatafora	Methanol	Against trypanosoma brucei	IC_50_ = 8.63 μg/ml	(Umeyama et al., 2014)
Anti-fibrotic	*C. cicadae* S.Z. Shing	Ergosterol peroxide	NRK-49 F cell line	Blockage of TGF-β1-stimulated phosphorylation of ERK1/2, p38 and JNK pathway, ↓ TGF-β1-induced fibroblasts	Zhu R et al. (2014)
Anti-atopic dermatitis	C. bassiana Z.Z. Li, C.R. Li, B. Huang and M.Z. Fan	Butanol fraction	Topical use of DNFB in NC/Nga mice	Blockade of histamine release, IgE production, IL-4, and IFN-γ secretion	Wu G et al. (2011)
Pro-apoptotic		4-Isopropyl-2-(1-phenylethyl) aniline	MDA-MB-231, HeLa, and C6 glioma cells	↓Proliferation of MDA-mb-231, HeLa, and C6 glioma cells, reduced the phosphorylation of STAT3, Src, and PI3K/p85	Kim M S et al. (2015)
Antitubercular	*Ophiocordyceps Communis* Hywel-Jones and Samson	Cordycommunin	*Mycobacterium tuberculosis* H37Ra	MIC = 15μM, weak cytotoxicity to kB cells	(Haritakun et al., 2010)
Anti-fatigue	*C. guangdongensis* T.H. Li, Q.Y. Lin and B. Song	Ethanol	Forced swimming	↓Blood lactic acid levels	(Yan et al., 2013)
Protein tyrosine phosphatase inhibitor	*C. gracilioides* Kobayasi	Terreusinone A, pinophilin C and cryptosporioptide A	PTP1B, SHP2, CDC25B, LAR and SHP1 enzyme	IC_50_ = 3.4–50 μg/ml	(Wei et al., 2015)
Antihyperlipidemic	*C. militaris* (L.) Fr.	Polysaccharides	HFD-induced	↓Blood and liver lipid, ↑SGPT, and antioxidant activity	Wang L et al. (2015)
Renoprotective	*C. pruinosa* Petch	Whole broth	LPS-induced renal cell injury	↓RBF and GFR, ED-1, GRP78, Beclin-1 autophagy and TUNEL apoptosis, ↑blood leukocyte counts, plasma blood urea nitrogen and creatinine level	Wu M F et al. (2011)
Anti-HIV-1		Cordysobin	HIV-1 reverse transcriptase	IC_50_ = 8.2 × 10^–3^ μM	Wang S X et al. (2012)
Renoprotective		Cyclosporine A	Cyclosporine-induced renal tubule dysfunction	↓Apoptosis, caspase-3 activation, ↑magnesium reabsorption channels TRMP6 and TRMP7	(Chyau et al., 2014)
Anti-asthmatic	*C. sphecocephala* (Klotzsch ex Berk.) Berk. and M.A. Curtis	Culture filtrate	Ovalbumin-induced asthmatic mice	↓IL-4, IL-13, and IL-25 expression and undesirable immune responses	(Heo et al., 2010)
Antiaging	*C. sinensis* (Berk.) Sacc.	Aqueous	D-galactose-induced aging	↑SOD, catalase, GSH, ↓MDA, monoamine oxidase	Chen et al. (1997)

### Anti-inflammatory Potential of Cordyceps spp.

The extract (ethanolic) of cultured mycelia of *C. militaris* (L.) Fr. possess potent anti-inflammatory activity in the carrageenin-triggered edema and decrement in inducible nitric oxide synthase (iNOS) expression in macrophages. Since the synthesis of NO by iNOS is elevated in inflammatory ailments and leads to cellular injury, this activity confirms its anti-inflammatory action (Won and Park, 2005). In lipopolysaccharide (LPS)-induced macrophage, NO production was restrained by butanolic fraction of *C. militaris* (L.) Fr. and the chief component was cordycepin. It was inferred that cordycepin inhibited the phosphorylation of protein kinase B (Akt), IκBα, and p38. It also suppressed TNF-α, cyclooxygenase-2 (COX-2), iNOS, and NF-κB translocation in these macrophages. Thus, hinted at the use of cordycepin for inflammation-linked disorders (Kim et al., 2006). *C. sinensis* has been reported to strengthen the cell-mediated immunity as well (Liu et al., 2007).

Interestingly, others have reported the application of *C. sinensis* as a cost-effective immunosuppressive agent after renal transplantation without obvious adverse effects (Li et al., 2009). Moreover, cordycepin and *C. sinensis* regulates the functions of human immune cells *in vitro* by promoting the expression of IL-1β, -6, -8, -10 and TNF-α of resting cells, and inhibited the phytohemagglutinin-induced expression of IL-2, -4, -5, -12 and IFN-γ and TNF-α. Furthermore, the cordycepin and *C. sinensis* treated human monocytic cell line (THP-1) exhibited a higher affinity for the transcription factors that are important in the gene regulation of various cytokines. Thus, cordycepin and *C. sinensis* regulates the immune cells via its immunoregulatory activity (Zhou et al., 2008). A heteropolysaccharide from cultured *C. sinensis* was reported to enhance the immunity in mice exposed to ionizing radiation by reducing oxidative injury and modulating the secretion of cytokines (IL-4, -5 and -17) (Zhang et al., 2011). It has been reported that methanolic fractions of *C. sinensis* contain ingredients having an immunosuppressive effect that inhibits blastogenesis, the activity of NK cell, and phytohemagglutinin induced IL-2 and TNF-α production by human mononuclear cells (Kuo et al., 1996). The crude extract and partially purified fractions of *C. sinensis* inhibit the generation of superoxide anion and release of elastase. Further, it was revealed that five constituents, cordysinins A-E accounted for these actions (Yang et al., 2011). The treatment of macrophages with diverse concentrations of *C. militaris* (L.) Fr. fruiting bodies (hot water extract) has potent suppressive effects on the production of these inflammatory mediators as evident by LPS-induced NO production, TNF-α, and IL-6 secretion (Jo et al., 2010).

Similar results were also reported in another study where the immune activation by CMP was improved. Moreover, CMP increased the thymus and spleen indices, the spleen lymphocyte activity, immunoglobulin G (IgG) function, and the total quantity of white blood cells in mice serum. CMP also enhanced the expression of IFN-γ, TNF-α, and IL-1β mRNA (Liu et al., 2016). Anti-inflammatory effects of another species of *Cordyceps* spp. i.e. *C*. *bassiana* Z.Z. Li, C.R. Li, B. Huang and M.Z. Fan was investigated (Kim T W et al., 2014). Its butanolic fraction showed the most effective anti-inflammatory response against LPS-activated RAW 264.7 macrophages by inhibiting IκB/NF-κB pathway and suppressing p38 and c-Jun N-terminal kinase (JNK) activation. Moreover, 4-quinolinol and 1-naphthol were found from *C. bassiana* as an anti-inflammatory compound.

*Paecilomyces hepiali* Q.T. Chen and R.Q. Dai, CBG-CS-2 strain, isolated from *Cordyceps* spp. was investigated for the anti-inflammatory effects (Park et al., 2014). It was documented that CBG-CS-2 downregulates the NO production, iNOS, and pro-inflammatory cytokines in LPS-stimulated macrophages by inhibition of NF-κB and activating protein (AP)-1, which are important in inflammation. Thus, the modulatory activity of CBG-CS-2 on the inflammatory response in macrophages, makes it useful as an anti-inflammatory drug or supplement. They further extended their study to confirm the immunoregulatory efficacy and safety of CBG-CS-2 separated and cultivated from *P. hepiali* from *C. sinensis* in healthy Korean adults (Jung et al., 2019). The major components reported i.e. CBG-CS-2, cordycepin, Polysaccharides, and adenosine induce immunomodulation by enhancing both the NK-cell activity and phagocyte reactions via macrophages activation. Moreover, cerebrosides have been reported to account for the anti-inflammatory activity of *C. militaris* (L.) Fr. namely cordycerebroside A, soyacerebroside I, and glucocerebroside (Chiu et al., 2016b). Summary of the factors involved in cordyceps-induced immunomodulatory and anti-inflammatory activity is depicted in Table 3. *C. sinensis* partially protected animal models of bacterial growth by activating macrophages. It can also induce the expression of IL-1β, IL-10, TNF-α, serum immunoglobulin IgG1, and IgG2b, as well as stimulates Th1 immune response using IFN-γ and IL-12 (Kuo et al., 2001; Lee et al., 2006). Concerning anti-inflammatory effects, cordymin, a purified compound from *C. sinensis* exhibited a decline in IL-1β, TNF-α, and pro-inflammatory markers in a carrageenan-induced inflammation model. Complementarily, the extracted compounds cordymin-1, cordymin-2, and cordymin-4 presented an antinociceptive effect in acetic acid-induced abdominal constrictions model (Qian et al., 2012). Similarly, the anti-inflammatory activity of *C. sinensis* extracts on the human neutrophils’ response was verified by inhibiting superoxide anion and elastase release. Most of the compounds produced an anti-inflammatory response superior to the indomethacin control, reaching a concentration necessary for 50% inhibition of 0.45 μg/ml for superoxide anion generation, and 1.68 μg/ml for elastase release. While for indomethacin, 38.32, and 31.98 μg/ml, respectively, were required (Yang et al., 2011). In another more detailed report, Cordycepin inhibited the overproduction of NO, prostaglandin E_2_, and pro-inflammatory cytokines in a dose-dependent manner on the production of inflammatory mediators in LPS-stimulated murine BV2 microglia. Those outcomes inferred that cordycepin has a high potential in restraining inflammatory mediators in neurodegenerative diseases (Jeong et al., 2010).

**TABLE 3:** Mechanism of action of the Cordyceps spp.-induced pharmacological activities.

Pharmacological activity	Mechanism of action	References
Immunomodulatory and anti-inflammatory activities	•Augmented the *in vivo* and *in vitro* NK activities phagocyte reactions via the activation of macrophages •Modulation of IL-6 production activated macrophages and enhance secretion of hematopoietic growth factors such as GM-CSF •Modulation of cytokines •Increase in an acid phosphatase activity, representing lysosomal enzymes, in macrophages •Inducing the expression of cytokines like IFN-γ, IL-12, and TNF- α •Increased TNF-α and IFN-γ, enhanced NO production, and induced iNOS mRNA and protein expressions in macrophage. Induction of mRNA expression of IL-1β, IL-6, IL-10, and TNF-α •Modulation of transcription factors involved in the gene regulation of various cytokines. •Upregulated the expression of TNF-α, IFN-γ, IL-6, and IL-1β •Stimulate NO production, phagocytosis, respiratory burst activity, secretion of IL-1β and IL-2 of macrophages •The decrement in iNOS expression in macrophages •Inhibited the phosphorylation of Akt, IκBα, and p38. Suppressed TNF-α, COX-2, iNOS, and translocation of NF-κB in macrophages •Inhibitory effects on the production of inflammatory mediators •Inhibition of IκB/NF-κB pathway and suppression of JNK and p38 activation •Suppresses the production NO, iNOS, and pro-inflammatory cytokines in macrophages via inhibition of NF-κB and AP-1	(Xu et al., 1992; Koh et al., 2002; Shin et al., 2003; Yu et al., 2004; Won and Park, 2005; Chen et al., 2006; Kim et al., 2006; Kuo et al., 2007; Ohta et al., 2007; Zhou et al., 2008; Zhu L et al. (2014); Kim T W et al. (2014); Park et al., 2014; Yang et al., 2015a; Chiu et al., 2016b; Liu et al., 2016; Jung et al., 2019)
Antioxidant and antiaging activity	•Inhibited MDA formation, anti-lipid peroxidation action and inhibited the accumulation of cholesteryl ester in macrophages •Attenuating the changes of GPx and SOD activities, inhibited MDA formation •Modulating antioxidation activity via significantly enhancing SOD activity of liver, brain, and serum as well as GPx activity of liver and brain in tumor-bearing mice •Improve the activity of SOD of RBCs, brain and liver, the activity of na^+^-K^+^-ATPE of the brain, the activity of catalase and GPx of blood, decrease the activity of monoamine oxidase of the brain and the contents of MDA of brain and liver in aged mice. •Improved the activity of SOD, glutathione peroxidase and catalase and lowered the level of lipid peroxidation and monoamine oxidase activity	(Yamaguchi et al., 2000a; Gu et al., 2003; Wang et al., 2004; Chen et al., 2006; Ji et al., 2009; Wang M et al. (2012)
Antitumor effects	•Via immunomodulation •Stimulating adenosine A3 receptors, Wnt signaling pathway, GSK3β activation cyclin D1 inhibition •Caspase activation and mitochondrial dysfunction •Via mTOR and AMPK signaling •Enhancing JNK and p38 kinase activity and activity of Bcl-2 pro-apoptotic molecules •Downregulating MDR/HIF-1α via AMPK/mTORC1 signaling •Antiangiogenic via inhibiting tube formation in endothelial cells and MMP reduction •Regulating Bcl-2 family and caspase activity and inhibition of COX-2 and prostaglandin E2 accumulation •Regulation of p85/Akt-dependent or GSK3β-related caspase-3-dependent apoptosis •Involvement of hedgehog, apoptosis, p53, and estrogen signaling	(Yoshida et al., 1989; Yoo et al., 2004; Park et al., 2005; Yoshikawa et al., 2007; Jin et al., 2008; Yoshikawa et al., 2008; He et al., 2010; Wong et al., 2010; Jen et al., 2011; Wu et al., 2014; Park et al., 2017; Lee et al., 2019)
Hypoglycemic activity	•Potentiated the activities of glucokinase, hexokinase and glucose-6-phosphate dehydrogenase •Increased the activity of hepatic glucokinase, the decline in the protein content of facilitative GLUT2 •By enhancing insulin sensitivity and improving oral glucose tolerance •Stimulates the expression of HNF-1α to activate GLUT2 for glucose uptake, induced AMPK phosphorylation, and gluconeogenesis inhibition •anti-PTP1B activity	(Kiho et al., 1996; Kiho et al., 1999; Balon et al., 2002; Zhao et al., 2002; Kim et al., 2017; Sun et al., 2019)
Hypocholesterolemic, hypotensive and vasorelaxation activities	•The endothelium-dependent vasorelaxant effect through stimulating the production of nitric oxide and endothelium-derived hyperpolarizing factor •anti-lipid peroxidation activities and inhibit the accumulation of cholesteryl ester in macrophages via suppression of LDL oxidation •Inhibiting LDL oxidation through scavenging free radicals •Increased the HDL cholesterol level, but decreased VLDL LDL cholesterol level •Inhibited PDGF-BB-induced RASMCs migration and proliferation via interfering with adenosine receptor-mediated NOS pathways •Reduced serum total cholesterol, triglyceride, LDL-C, VLDL-C as well as LDL-C/HDL-C and TC/HDL-C ratios. Increase in lipoprotein lipase (LPL) and hepatic lipase (HL) activity •Increase in levels of serum insulin •Reduction in the levels of blood and liver lipid, and improvement of the glutamate pyruvate transaminase and antioxidant activity	Yamaguchi et al., 2000a; Yamaguchi et al., 2000b; Chiou et al., 2000; Koh et al., 2003; Won et al., 2009; Gao et al., 2011; Guo et al., 2011; Wang L et al. (2015)
Anti-fatigue and antidepressant activity	•Facilitating efficient oxygen utilization, enhance energy metabolism in the mitochondria •The increasing level of *β-*ATP •Increased the metabolic threshold and the ventilatory threshold of the subjects •Extended the exhaustive swimming time of mice, hepatic and muscle glycogen levels, and decrease the blood lactic acid and blood urea nitrogen (BUN) levels •Upregulation of skeletal metabolic regulators AMPK, PGC-1 and PPAR- as well as activation of NRF-2-ARE pathway •Reducing the accumulation of blood lactic acid level. •Via decreasing MDA and 8-OHdG levels and increasing antioxidant enzymes activities (SOD, catalase and GPx) in the serum, liver and muscle of mice •Activating AMPK and protein kinase B (AKT)/mammalian target of rapamycin (mTOR) pathways and regulating serum hormone level	(Zhang et al., 1995; Xiao et al., 1999; Dai et al., 2001; Li and Li, 2009; Chen S et al. (2010); Kumar et al., 2011; Yan et al., 2012; Yan et al., 2013; Song et al., 2015; Geng et al., 2017)
Aphrodisiac potential	•PKC, cAMP-protein kinase A signal pathway •Induce the expression of steroidogenic acute regulatory (StAR) protein •Induce *in vivo* plasma corticosterone level •adenosine receptors activated cAMP-PKA-StAR pathway •PLC/PKC and MAPK signal transduction pathways •Stimulating CYP11A1, 3β-HSD, and CYP17A1 expressions	Wang et al. (1998), Huang et al. (2000), Hsu et al. (2003a), Hsu et al. (2003b), Huang B M et al. (2004), Chen et al. (2005), Leu et al. (2005), Chen S et al. (2010), Leu et al. (2011), Pao et al. (2012), Wang et al. (2016)

Footnote: DC-SIGN, dendritic cell-specific intercellular adhesion molecule-3-grabbing non-integrin; ERK, 1/2 extracellular signal-related kinases one and two; IL, interleukin; JNK, c-jun NH2-terminal kinase; MAPK, mitogen-activated protein kinase; MGL, macrophage galactose-type C-type lectin; MR, mannose receptor; MyD88, myeloid differentiation primary response protein 88; NF-κB, nuclear factor ‘kappa-light-chain-enhancer’ of activated B cells; P38, mitogen-activated protein kinase; PI3K, phosphatidylinositol 3-kinase; RAF, rapidly accelerated fibrosarcoma; RAS, rat sarcoma; SOCS, suppressor of cytokine signaling; SYK, Spleen tyrosine kinase; Th2, T helper type 2; TLR, toll-like receptor; Treg, regulatory T cells; TRIF, TIR domain-containing adapter inducing IFN-*β*.

### Antiviral effects of Cordyceps spp.

Intranasal administration of an acidic polysaccharide (APS), obtained from the extract of *C. militaris* (L.) Fr. cultivated on germinated soybeans, decreased the virus titers in the bronchoalveolar lavage fluid and the lung of mice infected with influenza A virus with increased survival rate. Furthermore, APS also increased TNF-α and IFN-γ levels. It enhanced NO production and induced iNOS mRNA and protein expressions in RAW 264.7 murine macrophage cells. The induction of mRNA expression of cytokines including IL-1β, IL-6, IL-10, and TNF-α demonstrated its beneficial therapeutic effects on influenza A virus infection by modulating immune function of macrophages (Ohta et al., 2007).

### Antioxidant and Antiaging Activity

The antioxidative profile of ethanol and water extract of *C. sinensis* (cultured) was assessed and initiated to be minimal on superoxide but it moderately inhibited MDA (malondialdehyde) formation (Yamaguchi et al., 2000a). *C. sinensis* has anti-lipid peroxidation potential and inhibits cholesteryl ester accumulation in macrophages thru LDL oxidation destruction. Li et al. (2001b) described that the adenosine content in *Cordyceps* spp. has no apparent relationship with anti-oxidation potential but later they confirmed that polysaccharides have the anti-oxidation profile.

Further, they extended their studies (Gu et al., 2003) and isolated a polysaccharide (210 kDa) from cultivated *Cordyceps* spp. mycelia having strong anti-oxidative activity. Thus, they concluded that *Cordyceps* spp. protects against neuronal cell toxicity. Chen et al. (Chen et al., 2006) informed that polysaccharide from *C. sinensis* probably inhibits tumor evolution mainly by modifying hosts' antioxidative action thru significantly enhancing SOD activity of brain, liver, and serum as well as GPx activity of liver and brain in tumor-bearing mice whereas, it remarkably reduces the MDA level in liver and brain (Chen et al., 2006).

Wu et al. performed an *in vitro* antioxidant activity of CM-hsCPS2 (a polysaccharide) which was isolated from fruiting bodies of *C. militaris* (L.) Fr. grownup on solid rice medium. (Wu F Y et al., 2011). Similarly, CBP-1 a novel polysaccharide was isolated from cultured *C. militaris* (L.) Fr. was testified to have the hydroxyl radical-scavenging power. Since these radicals are associated with the pathogenesis of several ailments, the study implicit for latent clinical applications of *C. militaris* (L.) Fr. as a substitute for *C. sinensis* in TCM (Yu et al., 2009).

Aging has been reported to involve oxidative stress by many researchers (Romano et al., 2010). A study by Wang et al., 2004 confirmed, that *C. sinensis* increases the capability of learning and memory, improve the action of SOD of RBC’s, mind and liver, the action of Na^+^-K^+^-ATPE of the brain, the potential of catalase and GPx of blood, and remarkably decline the activity of monoamine oxidase of the brain and the contents of MDA of brain and liver in aged mice by improving the antioxidative profile and eradicating free radicals (Wang et al., 2004). Ji et al. treated (Ji et al., 2009) D-galactose-induced senescence mice with *C. sinensis* extract. The results documented that *C. sinensis* extract can ameliorate the brain function and possess antioxidant activity by improving the activity of SOD, GPx, and catalase as well as lower the level of lipid peroxidation and monoamine oxidase. Another species i.e. *C. guangdongensis* has already been stated to have noteworthy antioxidative stress properties (Zeng et al., 2009). Another study with *C. guangdongensis* showed that it prolongs the mean lifespan and the half-death time of fruit flies in lifespan tests (Yan et al., 2011).

Structural and antioxidant analysis of W-CBP50, W-CBP50 I, and W-CBP50 II polysaccharides (from cultured *C. militaris* (L.) Fr.) was performed and all of them exhibited significant antioxidative strength (Chen X et al., 2013). Four polysaccharide fractions (CMP-1, CMP-2, CMP-3, and CMP-4) were extracted from cultured *C. militaris* (L.) Fr. depicted noticeable concentration-dependent antioxidant activities (Chen and Huang, 2014). Similarly, a novel low-molecular-weight polysaccharide (CMP-1) was isolated by Jing et al. (Jing et al., 2014) from the cultured *C. militaris* (L.) Fr. showed free radical-scavenging effects. The same group further isolated a novel polysaccharide (CMPA90–1; compound 1) from the cultured fruiting bodies of *C. militaris* (L.) Fr. that exhibited free-radical-scavenging effects (Jing et al., 2015). Summary of the factors involved in cordyceps-induced antioxidant and antiaging activity is depicted in Table 3.

### Antitumor Effects

Many genera of *Cordyceps* spp. (natural or cultured) has been documented to display the capability to restrain the growth of tumors due to various bioactive compounds present for e.g. polysaccharides, sterols, and adenosine (Yoshida et al., 1989; Bok et al., 1999; Li and Wang, 2008; Zhou et al., 2009). The glycosylated ergosterol from the methanolic extract of *C. sinensis* was reported as a remarkable antiproliferative compound against various tumor cell lines (Bok et al., 1999). Moreover, the water extract of *C. sinensis* also accelerates the Kupffer cells mediated phagocytosis to prevent metastasis (Nakamura et al., 1999). Since *Cordyceps* spp. can be cultivated artificially, it was documented in a comparative study that as compare to natural *Cordyceps* spp., the cultivated fungus has stronger antitumor activity against MCF-7, B16, HL-60, and HepG2 cancer cell lines (Zhang Q et al., 2005).

Cordycepin restrains the proliferation of cancer cells by triggering adenosine A3 receptors followed by the Wnt signaling pathway, including glycogen synthase kinase three beta (GSK3β) activation and cyclin D1 inhibition (Yoshikawa et al., 2004, 2007; Yoshikawa et al., 2008). In another study on MA-10 mouse Leydig tumor cell, cordycepin induced apoptosis was reported to involve caspase -9, 3, and -7 dependent pathway (Jen et al., 2011). Moreover, the antiproliferative response of cordycepin is documented to be mediated via the mammalian target of rapamycin (mTOR) and 5′AMP-activated protein kinase (AMPK) signaling (Wong et al., 2010). In human colorectal cancer cells, cordycepin triggers apoptosis via increasing B-cell lymphoma 2 (Bcl-2, proapoptotic molecules), JNK, and p38 kinase activity (He et al., 2010). As an adjuvant, a low concentration of cordycepin enhances the chemosensitivity of gall bladder cancer cells for gemcitabine and 5-fluorouracil, possibly via downregulating multiple drug-resistant/hypoxia-inducible factor 1 (MDR/HIF-1α) through regulating AMPK/mTORC1 signaling (Wu et al., 2014). Thus, it can be inferred that cordycepin induced antitumor profile involves plethora of pathways depending upon the cell type. Ji et al. reported the co-effect of fermented *C. sinensis* and selenium on uterine cervix cancer, where they reported that this combination attenuates the oxidative stress and refine the immune function as compared to their effect (Ji et al., 2014).

Aqueous extract from another species, *C. militaris* (L.) Fr*.* showed cytotoxic profile against stomach adenocarcinoma (SNU-1); colorectal adenocarcinoma (SUN-C4); and hepatocellular carcinoma (SNH-354), where cordycepin was reported as an active component (Lim et al., 2004). Extract of *C. militaris* (L.) Fr*.* possesses antiangiogenic properties as evident via inhibition of tube formation in endothelial cells and matrix metallopeptidase (MMP) reduction, a factor related to metastasis and invasion (Yoo et al., 2004). Similarly, *C. militaris* (L.) Fr*.* induces apoptosis via mitochondrial dysfunction and caspase activation in human breast cancer cell lines as well (Jin et al., 2008). Furthermore, pure compounds isolated from the extracts of *C. militaris* (L.) Fr*.* have been reported to be antiproliferative against PC-3, colon 205, and HepG2 cells (Rao et al., 2010). Furthermore, it was reported that *C. militaris* (L.) Fr*.* inhibit cancer growth through regulation of p85/Akt-dependent or GSK3β-related caspase-3-dependent apoptosis on a xenograft mouse model bearing murine T cell lymphoma (RMA) cell-derived cancers (Park et al., 2017).

It has also been documented that *C. sinensis* inhibits tumor-cell proliferation activities in different types of cancer cell lines, such as Jurkat, HepG2, PC 3, Colon 205, and MCF-7 (Rao et al., 2007). *C. militaris* (L.) Fr. concentrate and cordycepin elicit apoptosis via caspase-7, -8, and -9 involving the increase of Bcl-2-associated x protein (Bax)/Bcl-2 protein expression ratio and decreasing X-linked inhibitor of apoptosis protein (XIAP) thus confirming its anti-cancer property (Lee et al., 2019). Cordycepin exhibited an anti-cancer effect against B16 mouse melanoma by inducing the adenosine A3 receptor, and eventual activation of glycogen synthase kinase-3β, and the suppression of cyclin D_1_. Furthermore, cordycepin exerts a coadjuvant effect with other drugs, as demonstrated when combined with 2′-deoxycoformycin increased three hundred-fold the anti-cancer effect in B16 cells (Nakamura et al., 2015). Other mechanisms that describe the anti-cancer activity of *Cordyceps* spp. involve apoptosis and autophagy, as depicted in LNCaP (human prostate carcinoma) cells. Furthermore, the autophagy mechanism was evident by the increase and accumulation of microtubule-associated protein light chain-3 (LC3) (Lee et al., 2014). A summary of the factor involved in cordyceps-induced antitumor activity is depicted in Table 3.

### Hypoglycemic Activity

Kiho et al. (1993) displayed that polysaccharides obtained from the cultivated mycelium of *C. sinensis* (CS-F30) lower the plasma glucose level in normal and streptozotocin (STZ) induced diabetic mice by intraperitoneal administration in comparison to slight lowering through oral administration. Additionally, they also verified that CS-F30 potentiate the activities of glucokinase, hexokinase, and glucose-6-phosphate dehydrogenase thus accelerating the glucose metabolism, which in turn was responsible for its antidiabetic activity (Kiho et al., 1996). Kiho et al. (1993) also presented the intraperitoneal administration of CS-F10, a polysaccharide purified from hot water extract of cultured mycelium of *C. sinensis,* on normal, STZ-induced diabetic and epinephrine-induced hyperglycemic mice lowered the plasma glucose level and increased the activity of hepatic glucokinase. An industrial fermentation product i.e. CordyMax™ Cs-4 gained by a proprietary mycelial strain from natural *C. sinensis*, is described to be effective in lowering basal blood glucose and plasma insulin. Additionally, it improves the metabolism of glucose by increasing insulin sensitivity and improving oral glucose tolerance (Balon et al., 2002; Zhao et al., 2002). A study confirmed that the *Cordyceps* spp. has a hypoglycemic activity in nicotinamide (NA) and STZ-induced diabetic rats as evident by attenuation of the polydipsia, hyperglycemia, and weight loss (Lo et al., 2004). The extract from *C. sinensis* has been documented to promote *β*-cell survival in the diabetes mellitus-II mouse model. (Kan et al., 2012).

*C. sinensis* has already been stated to give a shielding effect on podocytes in rats with diabetic nephropathy (Hao et al., 2014). In the same way, CmNo1, a novel combination of the fruiting body and *C. militaris* (L.) Fr. mycelia, has also been testified to deliver renoprotection in high-fat diet and STZ–NA-induced diabetic (type 2) mice (Yu et al., 2016). Kim et al. in a study concluded that *C. militaris* (L.) Fr. water extract (CMW) stimulates the expression of hepatocyte nuclear factor (HNF)-1α to activate GLUT2 for glucose uptake in liver cells. Recently a study to isolate and characterize cerebrosides with anti- PTP1B activity from *C. militaris* (L.) Fr. was performed. The results documented that all four cerebrosides obtained from *C. militaris* (L.) Fr. exhibited inhibitory activity against PTP1B (Sun et al., 2019). A summary of the factors involved in cordyceps-induced hypoglycemic activity is depicted in Table 3.

### Hypocholesterolemic, Hypotensive and Vasorelaxation Activities

In previous studies, the existence of a protein component in *C. sinensis* is reported to diminish the mean arterial pressure of rats and induce a direct endothelium-dependent vasorelaxant effect through stimulating the production of NO and endothelium-derived hyperpolarizing factor. They reported the effect to be triggered by a single active constituent or by the combined action of many agents found in the extract that contributes to hypotensive and vasorelaxation activities (Chiou et al., 2000). Besides antioxidant profile, *C. sinensis* possess potent anti-lipid peroxidation activities and prevent the accumulation of cholesteryl ester in macrophages via suppression of LDL oxidation (Yamaguchi et al., 2000a).

Yamaguchi et al. (Yamaguchi et al., 2000b) performed the effect of the water extract from cultured CMW on serum lipid and lipid peroxide levels and aortic cholesterol accumulation using an atherosclerosis mouse model and concluded that CMW prevents cholesterol deposition in the aorta by impeding LDL oxidation via scavenging free radicals. Remarkably, a study was completed (Won et al., 2009) to determine the effect of cordycepin obtained by *C. militaris* (L.) Fr*.*, on responses of rat aortic smooth muscle cells (RASMCs) and vascular disorders, especially neointimal formation. It was documented that cordycepin inhibited platelet-derived growth factor-BB (PDGF-BB)-induced RASMCs migration and proliferation via interfering with adenosine receptor-mediated NOS pathways, thus resulting in the attenuation of neointima formation and thus could act as atherosclerosis agent. Furthermore, an increase in lipoprotein lipase (LPL) and hepatic lipase (HL) activity by cordycepin hint its contribution to lipid profiles regulation with no toxicity (Gao et al., 2011).

In a contemporary treatment approach toward both diabetes and depression management by vanadium-enriched *C. sinensis* (VECS). It was reported that in STZ-induced hyperglycemic rats administration of VECS, significantly reduces the blood glucose levels with the increase in levels of serum insulin (Guo et al., 2011). The study also revealed a remarkable decrease in immobility with a corresponding increase in the swimming and climbing behavior in hyperglycemic rats following VECS treatment thus concluding a contemporary treatment approach that advocates an aggressive stance toward both diabetes and depression management (Guo et al., 2011). Wang L et al. (2015) reported that the residual polysaccharide from *C. militaris* (L.) Fr. exhibited potential antihyperlipidemic, hepatoprotective, and antioxidant properties as depicted by the reduction in the levels of blood and liver lipid, and improvement of the glutamate pyruvate transaminase and antioxidant activity. Summary of the factors involved in *Cordyceps*-induced hypocholesterolemic, hypotensive and vasorelaxation activities is depicted in Table 3.

### Larvicidal Activity

Due to its eco-friendly nature and less or no side effects of microbial metabolites as an insecticide, they are of great use (Berdy, 1989). Kim et al. (2002) reported that *C. militaris* (L.) Fr. fruiting body-derived cordycepin acts as a naturally occurring insecticide against *Plutella xylostella* L. larvae via direct effect rather than an inhibitory action of chitin synthesis and that this compound has stomach action.

### Anti-fatigue and Antidepressant Activity

*Cordyceps* spp. has been used for ages as a medicine for increasing physical stamina to deal with weakness and fatigue by people of high altitude. *Cordyceps* spp. mushroom began to be in the spotlight in 1993, when some world athletics champions revealed part of their strategy for success, including a diet based on *Cordyceps* spp. ingredients (Kashyap et al., 2016). It works by an increase in cellular ATP increasing bioenergy and thus facilitating efficient oxygen utilization (Geng et al., 2017). Interestingly, athletes also use *Cordyceps* spp. to deal with fatigue and weakness thus increasing energy levels and extra endurance (Zhu et al., 1998). Dai et al. (2001) performed a study to evaluate the effects of CordyMax™ Cs-4, a mycelial fermentation product of *C. sinensis*, on energy metabolism. They documented that CordyMax remarkably improved the bioenergy status in the murine liver by increasing the level of *β-*ATP (adenosine triphosphate). Thus, the study supported the energy-promoting properties of CordyMax. As discussed above, the antioxidant properties of *Cordyceps* spp. enhance energy metabolism in the mitochondria and facilitate the efficient utilization of limited oxygen supply, thus increasing the anaerobic threshold (Zhang et al., 1995; Xiao et al., 1999). Since it has been well-established that fatigue is closely related to depression, a study was performed using the tail suspension test in mice to examine the antidepressant effects of supercritical fluid extract (SCCS) of *C. sinensis*. Results suggest that SCCS may elicit an antidepressant-like effect by affecting the adrenergic and dopaminergic systems, but not by affecting the serotonergic system (Nishizawa et al., 2007). To examine the effect of Cs-4 on erobic capacity in healthy elderly volunteers a double-blind, placebo-controlled trial was performed (Chen S et al., 2010). It was documented that administration of Cs-4 for 12 weeks, increased the metabolic threshold and the ventilatory threshold of the subjects. Such higher thresholds indicate better erobic performance without fatigue in older human subjects.

The effects of polysaccharides from *C. sinensis* mycelium on physical fatigue in mice documented that *C. sinensis* polysaccharides extended the exhaustive swimming time of mice, hepatic and muscle glycogen levels, and decrease the blood lactic acid and blood urea nitrogen (BUN) levels. Such observations confirmed the anti-fatigue effects of *C. sinensis* polysaccharides (Li and Li, 2009; Yan et al., 2012). To explore the underlying mechanisms behind the exercise endurance promoting activities of *C. sinensis,* Kumar et al. (2011) reported that such beneficial effects are mediated by upregulation of skeletal metabolic regulators AMPK, peroxisome proliferator-activated receptor gamma (PGC)-1 and peroxisome proliferator-activated receptors (PPAR)- as well as activation of NF-E2-related factor 2 (NRF-2)- antioxidant responsive element (ARE) pathway that reduces exercise-induced oxidative stress and inflammation. To other species, i.e. *C. guangdongensis* has also been reported that it exhibits anti-fatigue effect as evident by the longest swimming time in mice (Yan et al., 2011). Moreover, the active constituent accounting for *C. guangdongensis* induced anti-fatigue effect was reported to be a polysaccharide that alleviates fatigue by reducing the accumulation of blood lactic acid level (Yan et al., 2013).

Interestingly, it was reported that natural as well as laboratory cultured mycelia of *C. sinensis* can increase the motor coordination with improved metabolic and ventilatory that results in increased muscle endurance or antifatigue activity and mood elevator or antidepressant-like activity as a result of decreased endogenous depression (Singh et al., 2014). The antioxidative property of *C. sinensis* might be the reason for the increased skeletal muscle activity. Furthermore, *C. militaris* (L.) Fr. induces fatigue recovery is mainly through activating AMPK and AKT/mTOR pathways and regulating serum hormone level (Song et al., 2015). Summary of the factors involved in *Cordyceps*-induced anti-fatigue and antidepressant activity is depicted in Table 3.

### Aphrodisiac Potential

Because *Cordyceps* spp. is a benchmark for a highly energetic source, its applications as a sexual stimulant and in sexual dysfunction are attractive (Zhu et al., 1998; Tuli et al., 2013a; Chen et al., 2017), even popularly known as the Himalayan Viagra (Kashyap et al., 2016). *Cordyceps* spp. modulates the release of sexual hormones such as testosterone, estrogen, and progesterone, controlling reproductive activity, and restoring the impaired functions (Sohn et al., 2012). Mechanistically *Cordyceps* spp. stimulates steroidogenesis through PKA and PKC signal transduction pathways, testosterone production, and plasma testosterone levels, even in sexually inactive murine models (Huang et al., 2001; Chen et al., 2005). One study described that *C. sinensis* promoted prostate cancer cells grown in mice by enhancing testosterone production and androgen receptor expression (Ma et al., 2018). Hsu et al. (2003a) explored the effect of *C. sinensis* and its extracted fractions on testosterone secretion in mice using *in vivo* and *in vitro* approaches. Another research by Huang Y L et al. (2004) documented the effects of *C. sinensis* and its fractions on steroidogenesis in mice, where they inferred the remarkable stimulation of testosterone production.

In particular, the administration of cordycepin can increase the weight of the epididymis, sperm motility, and movement, and the number of mature sperm (Kashyap et al., 2016), namely, the quality and quantity of the sperm. Wang et al. (1998) demonstrate that PKC may be responsible for the *C. sinensis*-induced steroidogenesis in primary rat adrenal cell cultures. *C. sinensis* also triggers the steroidogenesis process in primary mouse Leydig cell and induces dose-dependent apoptosis in MA-10 mouse Leydig tumor cells (Leu et al., 2011; Pan et al., 2011). Moreover, *C. sinensis* has been reported by Huang et al. (2000) to induce the expression of steroidogenic acute regulatory (StAR) protein, a critical protein for steroidogenesis, in MA-10 mouse Leydig tumor cells. The same group further extended their studies (Huang et al., 2001) for *C. sinensis*-induced steroidogenesis in normal Leydig cells and reported that it had different effects on hCG-stimulated steroidogenesis between normal vs. tumor cells. They documented that *C. sinensis* significantly stimulated testosterone production and new protein synthesis was required for steroidogenesis (Huang et al., 2001). These results were also supported by others (Hsu et al., 2003a; Huang Y L et al., 2004) who documented that *C. sinensis* and its extracted fractions could stimulate testosterone production *in vitro* and *in vivo*. Hsu et al. (2003b) further explored the regulatory mechanism of action of *C. sinensis*-induced steroidogenesis using inhibitors of PKA or PKC pathways in normal mouse Leydig cells. Results documented that *C. sinensis* activated the cAMP-protein kinase A signal pathway, but not protein kinase C, and attenuated P450 side-chain cleavage enzyme (P450scc) activity to reduce human chorionic gonadotropin-stimulated steroidogenesis in purified mouse Leydig cells (Hsu et al., 2003b). However, Chen et al. Chen et al. (2005) reported that the mechanisms underlying *C. sinensis*-stimulated steroidogenesis in MA-10 mouse Leydig tumor cells possibly go through the PKA and PKC pathways simultaneously. They further explored the mechanisms of *C. sinensis*-stimulated steroidogenesis and found that *de novo* protein synthesis, increased steroidogenic acute regulatory protein mRNA expression, a calcium signal, and a mitochondria electrochemical gradient were required for *C. sinensis*-stimulated steroidogenesis (Chen S et al., 2010). Similarly, the effect of *C. sinensis* on the female reproductive system was also explored. It was showed that *C. sinensis* stimulates E2 production in human granulosa-lutein cells (GLCs) by upregulating the expression of several key enzymes, especially StAR and aromatase, making it a brilliant candidate for increasing the fecundity of women (Huang B M et al., 2004). Moreover, *C. sinensis* and its fractions have been reported to induce *in vivo* plasma corticosterone levels in immature and mature mice (Leu et al., 2005). Besides, *C. sinensis* could improve the function of reproduction and testis morphology in mice (Jin and Guo, 2006).

The *in vitro* effect of extracted fractions of *C. sinensis* mycelium on hCG-treated testosterone production from purified normal mouse Leydig cells was examined (Wong et al., 2007). It was reported that in normal mouse Leydig cells, all fractions of *C. sinensis* decreased hCG-stimulated testosterone production, which was opposite to the stimulatory effects of *C. sinensis* and fractions in tumor cells with hCG treatment. Different receptor subtypes between normal and tumor cells to activate different cellular functions are responsible for the difference (Huang et al., 1995; Huang et al., 1997). Administration of *C. militaris* (L.) Fr. mycelium improves sperm quality and quantity as evidenced by the improvement in the percentages of motile sperm cells and sperm morphology (Lin et al., 2007; Chang et al., 2008). Moreover, *in vivo* and *in vitro* effects of Cordycepin, were studied on primary mouse Leydig cell steroidogenesis. Cordycepin increased the plasma testosterone concentration as well as stimulated *in vitro* mouse Leydig cell testosterone production. It was reported that cordycepin associates with adenosine receptors to activate the cAMP-PKA-StAR pathway and steroidogenesis in the mouse Leydig cells (Leu et al., 2011).

Besides, others reported that cordycepin can stimulate progesterone production but also activate AR thus simultaneously induce steroidogenesis and apoptosis in MA-10 mouse Leydig tumor cells (Pan et al., 2011). Later intracellular phospholipase C/protein kinase C (PLC/PKC) and MAPK signal transduction pathways were reported to be responsible cordycepin induced steroidogenesis and cell death in MA-10 mouse Leydig tumor cells (Pao et al., 2012). However, long-term administration of cordycepin can counteract the decline of testicular function in middle-aged rats (Sohn et al., 2012). *C. militaris* (L.) Fr. remarkably protect testicles against oxidative damage caused by bisphenol A, a commonly used plasticizer, and relieved degeneration of serum T and LH concentration caused by it, via stimulating Star, CYP11A1, 3β-HSD, and CYP17A1 expressions (Wang et al., 2016). Summary of the factors involved in cordyceps-induced aphrodisiac potential is depicted in Table 3.

### Kidney Protection

The kidney is the main organ responsible for filtering and eliminating waste through the production of urine. Among the various applications of the components of *C. sinensis*, one can also find its valuable use to regulate some imbalances of the kidney, for example for the reduction of hematuria and proteinuria with an evident restoration of the tissue evidenced by histological analysis (Ding et al., 2011). In addition to supporting kidney transplantation in combination with drugs such as cyclosporin A. That combination is beneficial because high doses of cyclosporin A can induce kidney damage (Ding et al., 2009). Likewise, *C. sinensis* exhibits nephroprotection properties to mitigate damage from aminoglycosides, broad-spectrum antibiotics (Bao et al., 1994; Heggers et al., 1996). These properties are associated with an increase of 17-hydroxy-corticosteroid, 17-ketosteroid, SOD enzymes, and free radical scavenging.

## Toxic Effects of *Cordyceps* spp.

The array of secondary metabolites of polycyclic aromatic hydrocarbons (PAH) primarily developed from *C. sinensis* react with the polypropylene in popular bags, resulting in by-products toxic to *C. sinensis* and spectacular progress over time. These polypropylene/PAH by-products inevitably damage the organism. To extend the growth period of the organism, *C. sinensis* must be cultured in glass or metal vessels (Holliday et al., 2004). The PAH compounds are present in the living culture, but they are volatile compounds and lost after drying. While *Cordyceps* spp. cannot usually be grown in polypropylene bags, new strains that produce considerably less PAH are designed to allow them to grow in plastic bags.

## Conclusions and Prospects

Natural products are increasing the trust of people for the treatment and management of several chronic diseases. For hundreds of years, *Cordyceps* spp. have been used in Tibetan medicine and TCM, and in the last decades, the consumption of its fruiting bodies or related products as supplements has become popular. The most consumed and studied are *C. sinensis* and *C. militaris* (L.) Fr. *Cordyceps* spp. genus compromises a plethora of compounds and some of them showed therapeutic and pharmacological activities in pre-clinical studies, *in vitro,* and *in vivo*. Cordycepin and CA are important *Cordyceps* spp. bioactive constituents with important therapeutic applications associated with other compounds such as nucleotides, polysaccharides, cyclic peptides, sterols, and fatty acids are present in this genus and have shown a wide range of biological activities. The reservoir of *Cordyceps* spp. bioactive components show their therapeutic activities by modulating several cell signaling pathways due to the modulation of inflammation and oxidative/nitrosative stress processes. Cytokines releases, NO production via iNOS stimulation, and MAPK pathway are some of the cell signaling pathways modulated by *Cordyceps* spp. bioactive components. In the future, new chemical studies are needed to elucidate the unknown molecules present in *Cordyceps* spp., and new preclinical studies are needed to understand which compounds have the most interesting biological activities and the existing synergies between *Cordyceps* spp. components. Likewise, new drug formulations as nano drugs with cordycepin and other *Cordyceps* spp. biological compounds need to be developed and studied. However, new toxicological studies are needed to ensure their safety and promote its clinical studies. Clinical pilot studies with a few numbers of participants are needed as the first step to elucidate the potential of *Cordyceps* spp. as hypoglycaemic, hypocholesterolemic, and hypotensive agents. Other potential therapeutic effects such as anticancer may be more difficult to be elucidated in clinical studies and more pre-clinical studies are needed to a better understanding of the mechanisms involved. In conclusion, new future efforts are needed to elucidate the bioactive compounds present in *Cordyceps* genus and its therapeutic potential.
