# Correction: Cordyceps spp.: a review on its immune-stimulatory and other biological potentials

**DOI:** 10.3389/fphar.2026.1952854

**Published:** 2026-08-21

**Authors:** Gitishree Das, Han-Seung Shin, Gerardo Leyva-Gómez, María L. Del Prado-Audelo, Hernán Cortes, Yengkhom Disco Singh, Manasa Kumar Panda, Abhay Prakash Mishra, Manisha Nigam, Sarla Saklani, Praveen Kumar Chaturi, Miquel Martorell, Natália Cruz-Martins, Vineet Sharma, Neha Garg, Rohit Sharma, Jayanta Kumar Patra

**Affiliations:** 1 Research Institute of Biotechnology and Medical Converged Science, Dongguk University-Seoul, Goyangsi, South Korea; 2 Department of Food Science and Biotechnology, Dongguk University-Seoul, Goyangsi, South Korea; 3 Departamento de Farmacia, Facultad de Química, Universidad Nacional Autónoma de México, Ciudad de México, Mexico; 4 Laboratorio de Medicina Genómica, Departamento de Genética, Instituto Nacional de Rehabilitación Luis Guillermo Ibarra Ibarra, Ciudad de México, Mexico; 5 Department of Post-Harvest Technology, College of Horticulture and Forestry, Central Agricultural University, Pasighat, India; 6 Environment and Sustainability Department, CSIR-Institute of Minerals and Materials Technology, Bhubaneswar, India; 7 Adarsh Vijendra Institute of Pharmaceutical Sciences, Shobhit University, Saharanpur, India; 8 Department of Biochemistry, H. N. B. Garhwal University, Srinagar Garhwal, India; 9 Department of Pharmaceutical Chemistry, H. N. B. Garhwal University, Srinagar Garhwal, India; 10 Department of Nutrition and Dietetics, Faculty of Pharmacy, and Centre for Healthy Living, University of Concepción, Concepción, Chile; 11 Faculty of Medicine, Alameda Prof. Hernani Monteiro, University of Porto, Porto, Portugal; 12 Institute for Research and Innovation in Health, University of Porto, Porto, Portugal; 13 Laboratory of Neuropsychophysiology, Faculty of Psychology and Education Sciences, University of Porto, Porto, Portugal; 14 Department of Rasa Shastra and Bhaishajya Kalpana, Faculty of Ayurveda, Institute of Medical Sciences, Banaras Hindu University, Varanasi, India; 15 Department of Medicinal Chemistry, Faculty of Ayurveda, Institute of Medical Sciences, Banaras Hindu University, Varanasi, India

**Keywords:** ethnopharmacology, cordyceps, cordycepin, natural medicine, immune system, immunostimulation, immunomodulatory, clinical studies

Text correction

Original text: The presence of cordycepin (3′-deoxyadenosine) and 2′-deoxyadenosine in *C. sinensis* was characterized by using atomic attractive reverberation (NMR) and infrared spectroscopy (IR).

A correction has been made to the section [Chemical Compounds of Cordyceps spp.; Paragraph 1, Line number 9].

Corrected text: The presence of cordycepin (3′-deoxyadenosine) and 2′-deoxyadenosine in *C. sinensis* was characterized by using nuclear magnetic resonance (NMR) and infrared spectroscopy (IR).

The original version of this article has been updated.

